# Pediatric Difficult Airway Simulation Day

**DOI:** 10.5070/M5.52208

**Published:** 2026-01-31

**Authors:** Sarah Chen, Abha Athale, Anne Runkle

**Affiliations:** * Nationwide Children’s Hospital, Division of Emergency Medicine, Columbus, OH; ^ The Ohio State University, Department of Emergency Medicine, Columbus, OH

## Abstract

**Audience:**

This small-group simulation workshop is designed for pediatric emergency medicine fellows but can also be offered to emergency medicine residents or faculty.

**Introduction:**

Pediatric intubation is a high-acuity, low-frequency event. Specific patient scenarios that may lead to a difficult pediatric airway, such as airway edema, airway contamination (hemorrhage, emesis), prematurity, obesity, shock, and inhalational injuries, compound an already challenging and emergent situation. Previous studies have investigated simulation-based airway education for emergency medicine (EM), anesthesia, and critical care trainees. To our knowledge, there has been no study reporting the development and outcomes of a difficult airway course for pediatric emergency medicine (PEM) fellows covering emergency department (ED)-specific pediatric difficult airway content.

**Educational Objectives:**

The objective of this one-day simulation workshop is to increase learner confidence and skills necessary to perform critical pediatric airway procedures. PEM fellows of all training levels at our institution completed a three-hour “PEM Difficult Airway Day,” which consisted of six 30-minute stations focusing on airway scenarios critical for PEM fellow training: five high- and low-fidelity simulations (premature neonate, inhalational injury, contaminated airway, obese patient, and failed airway) and one discussion-based station on the physiologically difficult intubation. By the end of this workshop, learners will be able to: 1) identify various clinical situations in which a pediatric patient may have a difficult airway, 2) successfully intubate mannequins with simulated difficult airways using direct laryngoscopy (DL), video laryngoscopy (VL), laryngeal mask airway (LMA) placement, bougie-assisted intubation, and a hyper-angulated VL blade, and 3) recognize and describe the management of physiologically difficult airways and failed airways.

**Educational Methods:**

Small group activity combining procedural high- and low-fidelity simulations, as well as case-based learning.

**Research Methods:**

The PEM fellows completed pre- and post-workshop surveys to assess their airway knowledge and confidence regarding intubation using DL, VL, LMA placement, bougie-assisted intubation, intubation using a hyper-angulated VL blade, managing the anatomically difficult airway, managing the physiologically difficult airway, and managing the failed airway. In addition, learners were asked to identify any areas with continued knowledge gaps and low procedural confidence that they wished to be addressed in a future “PEM Difficult Airway Day.”

**Results:**

Our findings suggest that the “PEM Difficult Airway Day” significantly improved PEM fellow knowledge and confidence in infrequently performed critical pediatric EM scenarios, such as bougie-assisted intubation and use of a hyper-angulated VL blade, and knowledge of options and techniques for managing the anatomically difficult, physiologically difficult, and failed airway. There was no statistically significant improvement in confidence in DL and VL intubation and LMA placement. Additionally, fellows identified management of airway foreign bodies as an area with a continued knowledge gap and low procedural confidence.

**Discussion:**

A workshop dedicated to increasing the confidence and procedural skills necessary to perform critical airway procedures can be successfully offered to PEM fellows as a single-day, focused, small-group simulation workshop.

**Topics:**

Pediatric airway simulation, pediatric difficult airway, pediatric emergency medicine, simulation curriculum, medical education, workshop.

## USER GUIDE

**Table:** 

List of Resources:	
Abstract	73
User Guide	75
Instructor Materials	80
Appendix A: Small Group and Simulation Case Materials	80
Appendix B: Summary of List of Equipment Needed for All Scenarios	147
Appendix C: Pre-Survey	149
Appendix D: Post-Survey	154
Appendix E: Answer Key	160

**Learner Audience:**

These workshops are appropriate for interns, junior and senior residents, PEM Fellows, and EM/PEM faculty.

**Time Required for Implementation:** 3 hours total for all workshops

Instructor Preparation: 15 minutes set-up, 15 minutes tear-down

Time for case: 15–20 minutes

Time for debriefing: 15–20 minutes

**Recommended Number of Learners per Instructor:**

3–4

**Topics:**

Pediatric airway simulation, pediatric difficult airway, pediatric emergency medicine, simulation curriculum, medical education, workshop.

**Objectives:**

After completing this small-group activity, learners should be able to:

1. Identify various clinical situations in which a pediatric patient may have a difficult airway.
2. Successfully intubate mannequins with simulated difficult airways using direct laryngoscopy (DL), video laryngoscopy (VL), laryngeal mask airway (LMA) placement, bougie-assisted intubation, and a hyper-angulated VL blade.
3. Recognize and describe the management of physiologically difficult airways and failed airways.

### Linked objectives and methods

Pediatric intubation is a high-acuity, low-frequency event in comparison to adult intubation.^1^ These relatively rare incidences involve patients with unique anatomic and physiologic characteristics that render them vulnerable during airway management, and thus can make pediatric intubations particularly challenging.^2^ When compounded by specific scenarios that may lead to a difficult airway, an already critical situation may become even more dire. In fact, the multi-center registry PeDI (Pediatric Difficult Intubation) found that the most common severe complication of difficult pediatric tracheal intubations is cardiac arrest.^3^ These factors all illustrate the need for targeted training to improve provider confidence in critical airway management for pediatric patients.

Recent studies have investigated simulation-based airway education and various teaching methodology for mainly emergency medicine (EM),^4^,^5^ anesthesia,^6^,^7^ surgical,^8^–^10^ pediatric resident,^11^,^12^ and critical care^13^ trainees. Of these, some are focused on the difficult airway, but very few focused specifically on pediatric difficult airways.^9^,^11^,^12^,^14^–^16^ While there are two MedEdPORTAL articles currently published that detail a difficult airway course, neither focuses on EM-specific scenarios intended to teach management of difficult pediatric airway cases that trainees will be expected to manage independently in the emergency department (ED) without anesthesia or otolaryngology back-up on site.^13^,^16^ The setting for each simulation scenario in our workshop is a freestanding ED, with the goal of teaching pediatric emergency medicine (PEM) trainees to manage and stabilize pediatric patients with difficult airways prior to transfer to definitive care. This training is crucial for both PEM fellows and EM residents, as well as EM attendings who wish to maintain critical pediatric skills.

To our knowledge, no studies have reported the development and outcomes of a difficult airway course for PEM fellows covering EM-specific pediatric difficult airway content. Our objective was to assess PEM fellows’ baseline knowledge of difficult airway management techniques and confidence in performing critical procedures, to measure changes in knowledge and confidence after participating in a difficult airway course, and to identify persistent gaps in knowledge and procedural confidence.

The participants in the three-hour long PEM Difficult Airway Day included PEM fellows of all training levels, including EM residency-trained and pediatric residency-trained fellows. The day consisted of six 30-minute stations focusing on airway scenarios critical for PEM fellow training: five high- and low-fidelity simulations (premature neonate, inhalational injury, contaminated airway, obese patient, and failed airway) and one discussion-based station on the physiologically difficult intubation. This format was selected due to the emphasis on the practical application of the procedural skill being learned, specifically during difficult pediatric airway scenarios that would require the ED provider to secure the airway without immediate anesthesia or otolaryngology support. This helped achieve the goal of having learners identify various clinical situations in which a pediatric patient may have a difficult airway, successfully intubate mannequins with simulated difficult airways using DL, VL, LMA placement, bougie-assisted intubation, and a hyper-angulated VL blade, as well as recognize and describe the management of physiologically difficult airways and failed airways.

### Recommended pre-reading for instructor

Facilitator guide for each station includes debrief information.

### Associated content (optional)

Appendix A: Small Group and Simulation Case Materials

Appendix B: Summary of List of Equipment Needed for All Scenarios

Appendix C: Pre-survey

Appendix D: Post-survey

Appendix E: Answer Key

### Results and tips for successful implementation

The PEM Difficult Airway Day took place at a medical simulation center that had high- and low-fidelity airway mannequins, VL, Macintosh and Miller DL blades, LMAs, and bougies. The station leaders represented a variety of subspecialties, including PEM, EM, and Neonatal Intensive Care Unit (NICU) physicians. To provide a VL for each station, we obtained additional devices by reaching out to our hospital’s VL device representative as well as our ED education coordinator.

The PEM fellows rotated through six 30-minute stations focusing on airway scenarios critical for PEM fellow training:

1. **Premature neonate:** This simulation station consisted of a 31-week gestation (1400 g) premature infant who was delivered emergently in the ED with respiratory failure. The learners were taught how to select intubation supplies (blade size, endotracheal tube size) and induction agents. The learners then practiced DL intubations on the high-fidelity premature infant mannequin.
2. **Inhalational injury:** This simulation station consisted of a burn patient with inhalational injury. The learners were taught indications for intubating burn victims and potential anatomic and physiologic difficulties these patients may pose during intubation. The learners were able to practice DL, VL, and bougie-assisted intubations on a low-fidelity mannequin.
3. **Contaminated airway:** This simulation station consisted of a patient with intractable emesis and respiratory failure. The learners were taught the SALAD (Suction-Assisted Laryngoscopy and Airway Decontamination) technique and were given the opportunity to practice intubating a low-fidelity mannequin that would have profuse “emesis” consisting of a mixture of water, green food coloring, and xanthan gum that was pumped through the mannequin’s esophagus using an improvised pump mechanism.
4. **Obese patient:** This simulation station consisted of an obese patient with respiratory failure. The learners were taught the anatomic and physiologic difficulties of intubating an obese patient, and were able to practice DL, VL, and bougie-assisted intubations on a low-fidelity mannequin.
5. **Failed airway:** This station consisted of a status epilepticus patient with respiratory failure and a difficult airway due to a tongue injury and intraoral bleeding. The learners reviewed the failed airway algorithm and were taught various airway rescue techniques including oropharyngeal airway (OPA), LMA, bougie, and hyper-angulated blade.
6. **Physiologically difficult airway:** This was a discussion-based, flipped-classroom-styled station about physiologically difficult intubations. Learners explored cases including refractory hypoxia, septic shock, metabolic acidosis, status asthmaticus, and the use of delayed sequence intubation in an agitated patient with hypoxia.

The PEM fellows completed an IRB-approved pre- and post-course survey (Appendix C and D) assessing their airway knowledge and confidence. The survey, adapted from previous simulation-based intubation educational studies,^17^,^18^ asked fellows to rate their ability to perform each of the following tasks: intubation using DL, VL, LMA placement, bougie-assisted intubation, intubation using a hyper-angulated VL blade, managing the anatomically difficult airway, managing the physiologically difficult airway, and managing the failed airway. The answer choices were based on a 4-point Likert scale: 1= could not perform, 2= may be able to perform with assistance, 3= may be able to perform independently, 4= easily able to perform independently. All surveys were de-identified and administered via REDCap, and pre- and post-course survey responses were compared with a two-tailed paired t-test.

Thirteen survey responses were collected from the PEM fellows who participated in the course. There was no statistical difference (defined as p-value <0.05) between confidence in performing intubation with DL, intubation with VL, or LMA placement before and after the course. In contrast, there was a significant increase in confidence in intubation with a hyper-angulated VL blade (p-value= 0.01), bougie-assisted intubation (p-value = 0.001), management of the anatomically difficult airway (p-value= 0.005), management of the physiologically difficult airway (p-value= 0.0001), and management of the failed airway (p-value= 0.002). Additionally, fellows identified management of airway foreign bodies as an area with a continued knowledge gap and low procedural confidence.

Our findings suggest that the “PEM Difficult Airway Day” significantly improved learner knowledge and confidence in infrequently performed critical pediatric EM scenarios, such as bougie-assisted intubation and use of a hyper-angulated VL blade, and in knowledge of options and techniques for managing the anatomically difficult, physiologically difficult, and failed airway. While there was not significant improvement in confidence in DL and VL intubation and LMA placement, we postulate this is likely due to higher baseline comfort with these more common procedures. We are developing an airway foreign body simulation for the next course and will be adapting the course for PEM faculty and EM residents. While the trainees in this study were PEM fellows, this difficult airway day model could easily be modified for educating EM residents and attendings who would like to maintain crucial pediatric skills. Pediatric airway management is a critical procedure that EM residents perform infrequently and report low confidence in their ability to perform.^19^ This course provides an opportunity for trainees to gain knowledge and confidence in managing the difficult pediatric airway in a simulated environment.

## Figures and Tables

**Table:** SIMULATION EVENTS TABLE:

Minute (state)	Participant action/trigger	Patient status (simulator response) & operator prompts	Monitor display (vital signs)
0:00 (State 1: Respiratory Distress)	Continue PPV Perform steps of MR SOPA Verbalize intent to intubate without RSI medications	**Operator notes/prompts:** There are two facilitators. One can be an RT performing PPV via T-Piece Resuscitator, while the trainee holds the jaw thrust and seal. The other facilitator can obtain the equipment/medications requested by the trainee, answer questions, and prompt. If the trainees do not attempt MR SOPA or if they start chest compressions without establishing an advanced airway, stop the simulation, debrief, and restart simulation.	HR: 70 bpm RR: 40 breaths/min (PPV) SpO_2_: 75% (PPV)
1:00	Prepare for intubation by obtaining airway equipment and positioning the patient	**Operator notes/prompts:** If the trainee chooses to intubate with RSI (incorrect choice), it will take 60 seconds to obtain the medications and 45 seconds for the medications to take effect; meanwhile, the patient will become bradycardic (go to state 3). If they start compressions prior to intubation, remind the trainee that NRP requires establishing an advanced airway prior to compressions. If the trainees accidentally intubate the esophagus, after 15 seconds the patient will desaturate and become bradycardic (go to state 3).	HR: 70 bpm RR: 40 breaths/min (PPV) SpO_2_: 75% (PPV)
2:00	Intubate the patient in a <30 second attempt with 3.0 uncuffed endotracheal tube (ETT) BURP (backwards, upwards, rightwards pressure) maneuver for troubleshooting If successful, go to state 2	**Operator notes/prompts:** “What laryngoscope blade and ETT do you want? How do you want your stylet bent?” and hand to trainee. If attempting to intubate with an ETT size >3.0, the participant will not succeed. Case ends. If they intubate with 2.5 or 2.0 ETT, discuss in the debrief.	HR: 70 bpm RR: 40 breaths/min (PPV) SpO_2_: 75% (PPV)
2:30 (State 2: Successful Intubation)	Call NICU for admission (can discuss SpO2 goal and vent settings) Confirm bilateral breath sounds Request postintubation chest x-ray (can verbalize that it shows signs of RDS) Request postintubation sedation (can discuss in debrief) Consider causes of respiratory failure in the neonate (not the point of this simulation – focus on airway management)	**Operator notes/prompts:** After brief discussion of next steps, move to debrief section. This completes the case.	HR: 130 bpm RR: 40 breaths/min. on the vent BP: 50/28 (35) SpO_2_: 100% on 100% FiO_2_
Alternate 2:30 (State 3: Bradycardia)	If trainees ask for RSI medications, and do not start intubation by the 2-minute mark without establishing an advanced airway, or if they accidentally intubate the esophagus, after 15 seconds the patient will desaturate and become bradycardic	**Operator notes/prompt:** Prompt trainees to restart PPV, if they have not immediately started. If trainees asked for RSI medications, say “your nurse is still going to find them.” If they keep asking for RSI meds, this ends the case. If esophageal intubation or unsuccessful intubation, have trainees verbalize how they will change their approach for their second intubation attempt. Allow only one additional intubation attempt. If trainees successfully intubate, go to State 2, otherwise, end the case.	HR: 40 bpm RR: 40 breaths if giving PPV, otherwise gasping, irregular breaths BP: 50/28 (35) SpO_2_: 60% SpO2 can drift up to 75% after PPV starts. Heart rate improves to 80 bpm

**Figure:**
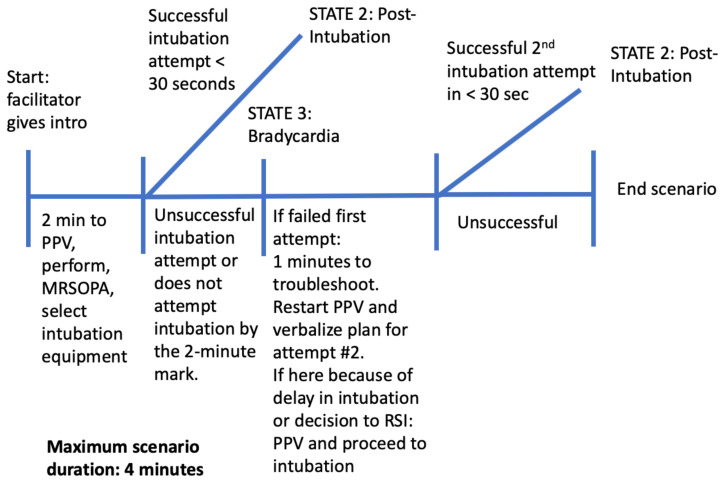


**Table:** 

**Critical Actions:** Start PPV (21–30% FiO2, PEEP 5, PIP 20) MR SOPA steps Successful intubation with appropriately sized intubation equipment and without RSI medications	0:00

**Table:** 

Weight (gm)	Gestational Age	ETT size (internal diameter in mm)	Blade Size
< 1000	< 28 weeks	2.5	00 or 0
1000–2000	28–34 weeks	3.0	0
2000–3000	24–38 weeks	3.5	0
> 3000	> 38 weeks	3.5 or 4.0 (if LGA)	0 or 1

**Table:** 

**Vitals:**	Heart Rate (HR) 130	Blood Pressure (BP) 130/80	Respiratory Rate (RR) 24
	Temperature (T) 37°C	Oxygen Saturation (O_2_Sat) 86%	

**Table:** SIMULATION EVENTS TABLE:

Minute (state)	Participant action/trigger	Patient status (simulator response) & operator prompts	Monitor display (vital signs)
0:00 (State 1)	Ask for RSI meds and intubation equipment Preoxygenate with NC and NRB, or HFNC Ramp/position patient If correct patient positioning AND adequate preoxygenation AND intubation attempt is < 30 seconds, scenario successfully ends (go to state 2)	**Operator notes/prompts:** Prompt with “Do we need to intubate him?” if not proceeding towards intubation within 60 seconds. Improve SpO2 to 96% if placed on NC/NRB or HFNC. If placed on suboptimal preoxygenation or patient is not ramped, SpO2 90% (go to state 3). Once the patient is preoxygenated and equipment obtained, can “fast forward” by asking the trainees how long they want to preoxygenate and skip to pushing RSI. Make sure they wait 30 seconds for RSI to take effect.	T: 37°C HR: 130 bpm RR: 24 breaths/minute BP: 130/80 SpO_2_: 86% on room air
4:00 (State 2: Successful Intubation)	Keep patient on 100% FiO2 Confirm bilateral breath sounds Request postintubation sedation and CXR Admit to PICU	**Operator notes/prompts:** End scenario as soon as patient intubated successfully and they order sedation and CXR.	T: 37°C HR: 100 bpm RR: 12 breaths/minute (ventilator) BP: 130/80 SpO_2_: 99% on 100% FiO2
Alternate 4:00 (State 3: Hypoxia)	If improper positioning, inadequate preoxygenation, or intubation attempt >30 seconds, patient desaturates to 65%	**Operator notes/prompts:** If the trainee goes right to intubation (does not bag up with BVM or LMA) and does not get the ETT in 30 seconds, use BVM or LMA but second intubation attempt takes > 30 seconds, or spend >2 minutes bagging, say, “Your patient desaturates more and goes into pulseless electrical activity (PEA) arrest. This ends the scenario.” Increase SpO2 to 70% if single provider is bagging. If asked, there is minimal chest rise, and difficulty bagging. Increase SpO2 to 80% if 2 person BVM with good technique. Increase to 88% with LMA placement. If trainees successfully intubate in <30 seconds, go to State 2.	T: 37°C HR: 100 bpm RR: 0 (apneic after RSI) BP: 130/80 SpO_2_: 65%

**Figure:**
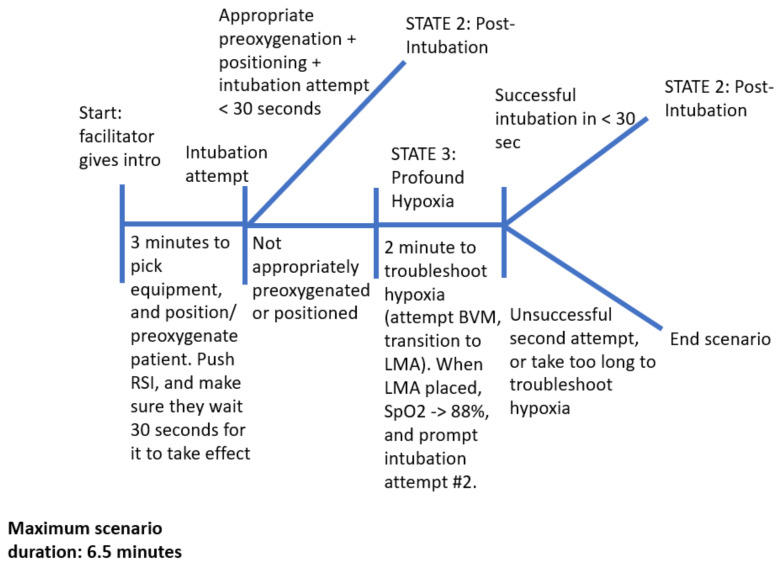


**Table:** 

**Critical Actions:** Preoxygenate the patient while preparing for intubation. Ramp the patient with pillows/blankets underneath to optimize positioning prior to intubation.	0:00

**Table:** 

**Vitals:**	Heart Rate (HR) 145	Blood Pressure (BP) 100/76	Respiratory Rate (RR) 50
	Oxygen Saturation (O_2_Sat) 90% on room air		

**Table:** SIMULATION EVENTS TABLE:

Minute (state)	Participant action/trigger	Patient status (simulator response) & operator prompts	Monitor display (vital signs)
0:00 (State 1: Preparation)	Recognize need for advanced airway prior to arrival and begin setup. Gather intubation supplies and medications (ketamine and rocuronium will be immediately ready) within 90 seconds. Perform primary survey, stopping at “Airway.” Recognize respiratory distress and start supplemental O2 (NRB mask set to >10 LPM) + NC at flush rate. Could also do HFNC if ready by the time EMS arrives (unlikely to happen).	**Operator notes/prompts:** Read the prompt, then have patient “arrive” via EMS 90 seconds after initial prompt by uncovering sheet over manikin. If no supplemental O2 started by 90 seconds from uncovering manikin, have the patient desaturate to 85% SpO2. If no immediate intervention (within 30 seconds), the patient becomes obtunded and desaturates to 70%. Facilitator can prompt them to notice the hypoxia – if no immediate intervention, end the scenario and restart.	HR: 145 bpm RR: 50 breaths/min BP: 100/76 SpO_2_: 90% on room air
3:00 Intubation	Perform proper preoxygenation, complete intubation within 5 minutes of scenario start time, and intubation duration =< 30 seconds. If successful, go to state 2	Can intubate with 4.0–5.0 ETT. If trainee tries to intubate with a > 5.0 ETT, they will not be able to pass the tube due to airway edema (go to state 3). If the participant tries to intubate with an ETT <4.0, there will be a large air leak and oxygenation will not improve (go to state 3). ○ Will need to verbalize what they will do differently than on first attempt. If they fail their first intubation attempt or it takes > 30 seconds, go to state 3.	HR: 100 bpm RR: 50 breaths/min (post RSI, RR = 0) BP: 92/68 SpO_2_: 70%
4:15 Post-Intubation)	Keep patient on 100% FiO2 Confirm bilateral breath sounds Request postintubation sedation and CXR Admit to PICU		HR: 100 bpm RR: 20 breaths/min (on ventilator) BP: 92/68 SpO_2_: 92%
Alternate 4:15 (State 3: Hypoxia)	They can arrive at this state after a failed or prolonged intubation attempt. Critical actions: troubleshoot hypoxia via LMA, BVM, attempt reintubation	**Operator notes/prompts:** If they attempt BVM, even with 2-person seal, poor ventilation, no improvement in SpO2. If they place LMA <60 seconds after failed first intubation attempt, patient will show improved ventilation, SpO2 85%. Can make this a near-immediate improvement (to speed the scenario) If they do not place LMA within 60 seconds, end the scenario, stating the patient becomes more hypoxic, bradycardic, and loses pulses. If they place LMA within 60 seconds, but then get stuck on what to do next, you can end the scenario as well. If they are successful on second intubation attempt, go to state 2. If unsuccessful or prolonged attempt > 30 seconds, end the scenario.	HR: 60 bpm RR: 0 breaths/min BP: undetectable SpO_2_: 70%

**Figure:**
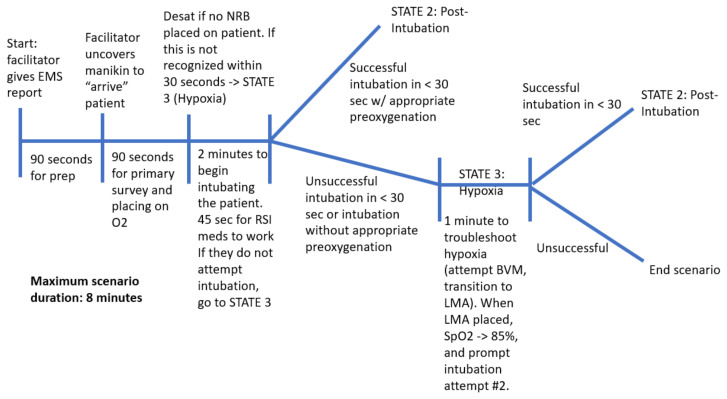


**Table:** 

**Critical Actions:** Immediate preoxygenation and recognition that the patient will require an advanced airway. Intubation with appropriately sized airway equipment, considering that the airway may be more edematous. Utilize LMA quickly as a temporizing airway if needed.	0:00

**Table:** 

**Vitals:**	Heart Rate (HR) 90	Blood Pressure (BP) 140/70	Respiratory Rate (RR) 12
	Oxygen Saturation (O_2_Sat) 95% on NRB		

**Table:** SIMULATION EVENTS TABLE:

Minute (state)	Participant action/trigger	Patient status (simulator response) & operator prompts	Monitor display (vital signs)
0:00 (State 1: Preparation)	Choose intubation supplies Position patient (can do flat or head of bed elevated) Ask for double suction and test suction	**Operator notes/prompts:** Facilitator can prompt **“**Where**’**s the suction**?”** if trainee does not check for suction. DO NOT ALLOW THEM TO WAIT/DELAY – Facilitator/nurse will push RSI at the 90 second mark no matter what.	T : Unavailable HR : 90 bpm RR : 12 BP: 140/ 70 SpO_2_: 95% on NRB at 100%
1:30	If they intubate successfully, go to state 2	**Operator notes/prompts:** Facilitator will activate profuse emesis mechanism 15 seconds after RSI medications pushed by facilitator. If patient is not successfully intubated in under 30 seconds after emesis begins, patient becomes profoundly hypoxic (go to state 3).	
2:15 (State 2: Post-Intubation)	Successfully intubated through emesis within 30 seconds using double suction Keep patient on 100% FiO2 Confirm bilateral breath sounds Request post-intubation sedation and CXR Admit to PICU	**Operator notes/prompts:** End scenario as soon as patient intubated successfully and they order sedation and CXR.	HR: 80 bpm RR: 12 (on vent) BP: 140/70 SpO_2_: 92% (on vent)
Alternate 2:15 (State 3: Hypoxia)	Unable to successfully intubate through emesis within 30 seconds	**Operator notes/prompts:** If unable to intubate in <30 seconds, patient becomes profoundly hypoxic and arrests. End scenario promptly after the failed intubation, and, after debriefing, run the scenario again.	HR: 100 bpm (PEA) RR: 0 BP: 80/70 SpO_2_: 60%

**Figure:**
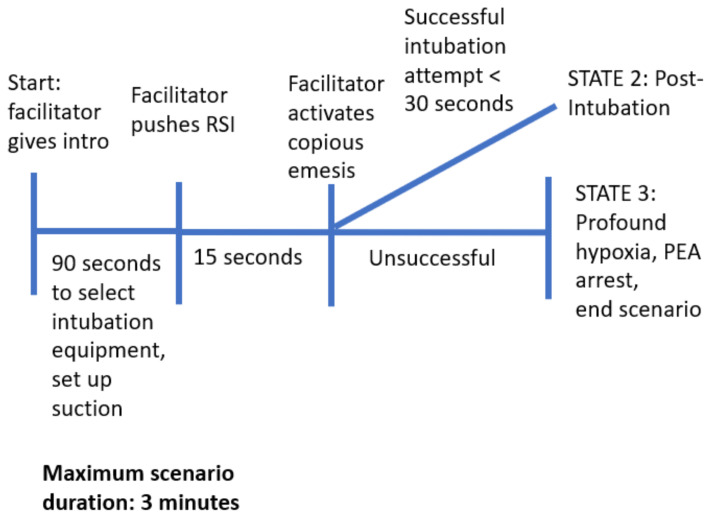


**Table:** 

**Critical Actions:** Have multiple suction devices prepared (Yankauer x2) when intubating contaminated airways Use appropriate suctioning technique to successfully intubate the patient with large volume emesis.	0:00

**Table:** 

**Vitals:** Heart Rate (HR) 150	Blood Pressure (BP) 100/60
Respiratory Rate (RR) 20 (BVM Ventilation	Temperature (T) 102.5**°**F
Oxygen Saturation (O_2_Sat) 93%	

**Table:** SIMULATION EVENTS TABLE:

Minute (state)	Participant action/trigger	Patient status (simulator response) & operator prompts	Monitor display (vital signs)
0:00 (State 1)	Select intubation equipment after prompt is read	**Operator notes/prompts:** Learners must choose intubation equipment – CMAC Miller 1/Mac 2 and 3.5 and 4.0 ETT will be on counter. If asked about blood around mouth, facilitator can suggest that patient may have bitten his tongue. Facilitator will push RSI 60 seconds after starting scenario (tell participant that you are going ahead); allow 45 seconds for RSI medications to take effect. Facilitator to prompt **“**Hey, what**’**s your backup plan**?”** when pushing RSI.	T: 102.5**°**F HR: 150 bpm RR: 20 (being bagged) BP: 100/60 SpO_2_: 93% on 100% FiO2 via BVM
1:45	Attempt intubation If unable to intubate <30 seconds, cease attempts, recognize failed airway, and start bagging/rescue maneuvers	**Operator notes/prompts:** If successfully intubated in < 30 seconds, go to state 2. If not successfully intubated <30 seconds after beginning attempt, allow trainees to try again. ○ Go to state 3.	
2:15 (State 2: Post-Intubation)	Keep patient on 100% FiO2 Confirm bilateral breath sounds Request post-intubation sedation and CXR Admit to PICU	**Operator notes/prompts:** Can have O2 slowly trend up to 95%. If you notice that all your groups are getting the intubation on the first try, you can make the following modification: the ETT comes out while RT is securing it and the patient becomes hypoxic (go to state 3), forcing trainees to bag and place an LMA.	T: 102.5**°**F HR: 150 bpm RR: 20 (being bagged) BP: 100/60 SpO_2_: 93% on 100% FiO2 via BVM
Alternate 2:15 (State 3: Hypoxia)	Recognize failed airway Place OPA or LMA Verbalize what will be done differently for second attempt	**Operator notes/prompts:** If no quick intervention, drop SpO2 to 70%, then state that the patient had a bradycardic PEA arrest and end scenario. If bagging with BVM-> O2 goes to 72%. If OPA tried, O2 -> 80%. LMA -> O2 goes to 86%. Prompt them to verbalize what they will do differently on their second attempt. Give them 2 minutes to troubleshoot the failed airway/hypoxia. If they do not attempt reintubation in 2 minutes, end the scenario.	T: 102.5**°**F HR: 50 bpm RR: 20 (on vent) BP: 80/40 SpO_2_: 70%
4:15	Perform second intubation attempt	**Operator notes/prompts:** If they successfully intubate in under 30 seconds, go to state 2. If not, end the scenario.	

**Figure:**
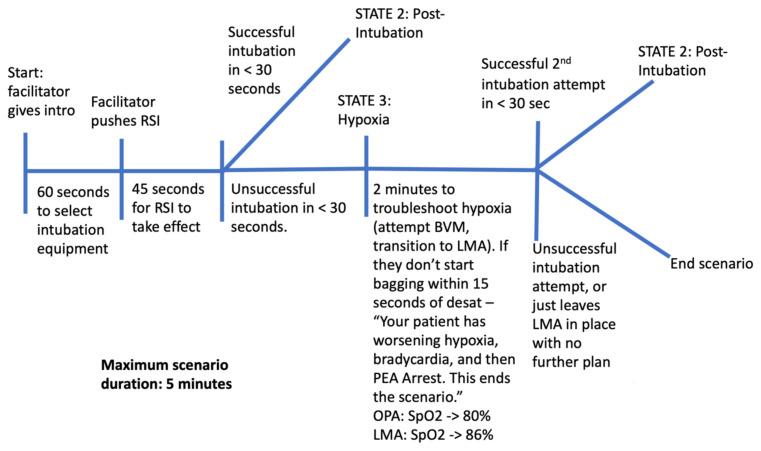


**Table:** 

**Critical Actions:** 1. Recognize a failed airway. 2. Be able to utilize airway adjuncts in a failed airway scenario.	0:00

**Figure:**
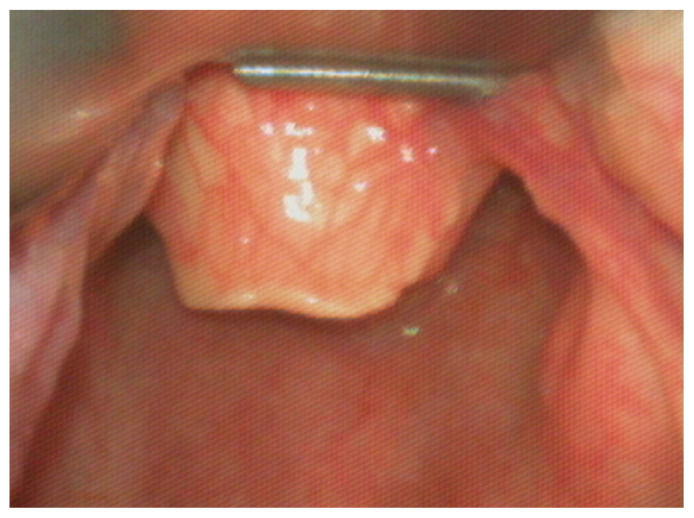
Source: Hofmeyr, R. Coremack-Lehane Grading Examples. In: Open Airway. https://openairway.org/cormack-lehane-grading-examples/. Accessed November 19, 2025. CC-BY-NC-AS

**Figure:**
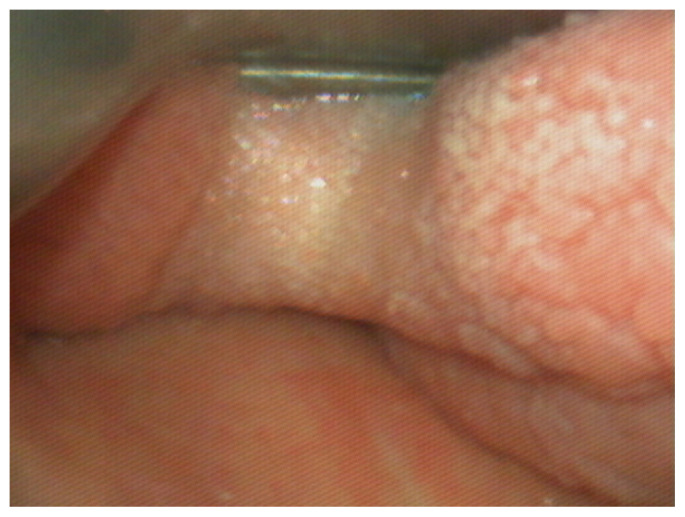
Source: Hofmeyr, R. Coremack-Lehane Grading Examples. In: Open Airway. https://openairway.org/cormack-lehane-grading-examples/. Accessed November 19, 2025. CC-BY-NC-AS

**Figure:**
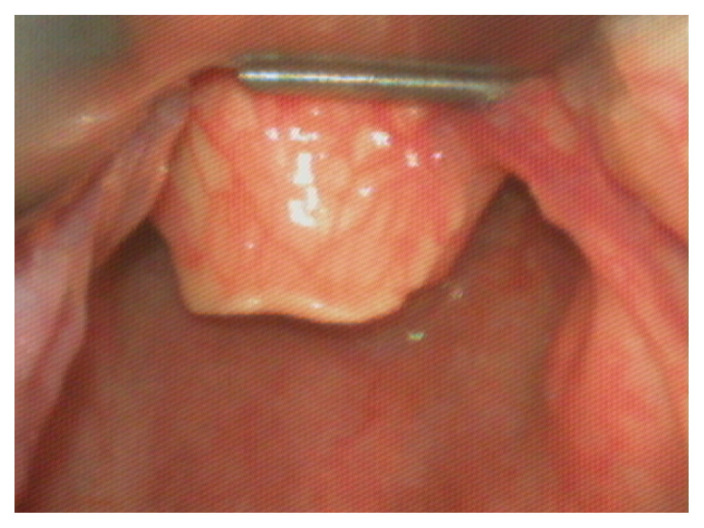
Source: Hofmeyr, R. Coremack-Lehane Grading Examples. In: Open Airway. https://openairway.org/cormack-lehane-grading-examples/. Accessed November 19, 2025. CC-BY-NC-AS

**Figure:**
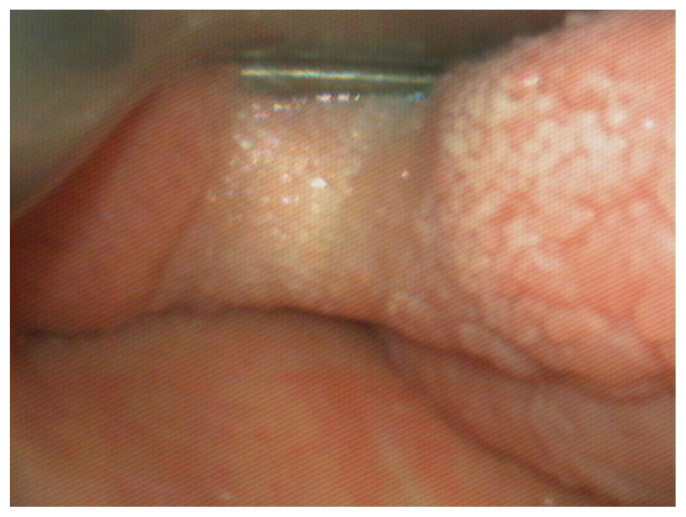
Source: Hofmeyr, R. Coremack-Lehane Grading Examples. In: Open Airway. https://openairway.org/cormack-lehane-grading-examples/. Accessed November 19, 2025. CC-BY-NC-AS

**Figure:**
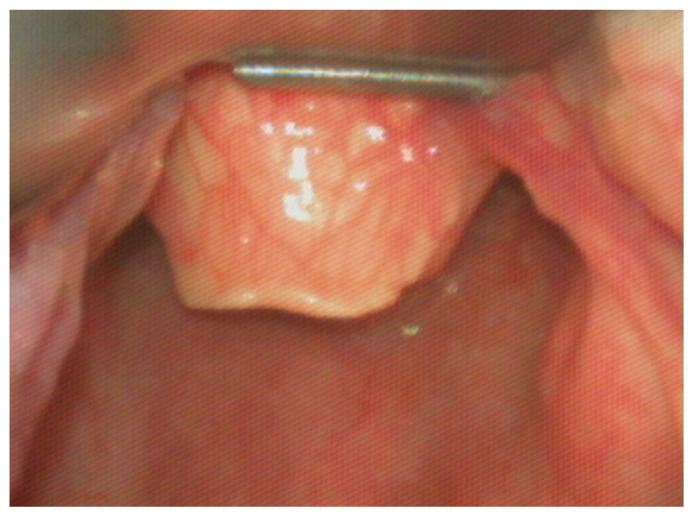
Source: Hofmeyr, R. Coremack-Lehane Grading Examples. In: Open Airway. https://openairway.org/cormack-lehane-grading-examples/. Accessed November 19, 2025. CC-BY-NC-AS

## INSTRUCTOR MATERIALS

### Appendix A Small Group and Simulation Case Materials

Station 1: Premature Neonate Airway

Station 2: Obese Patient

Station 3: Inhalational Injury

Station 4: Contaminated Airway

Station 5: Failed Airway

Station 6: Physiologically Difficulty Intubation

Pediatric Difficult Airway Day Clinical Pearls

*Station 1: Premature Neonate Airway*

**Case Title:** Premature Neonate Airway

**Case Description & Diagnosis (short synopsis):** A premature male was born precipitously outside your freestanding ED. He was found to have Respiratory Distress Syndrome (RDS) and ultimately needed endotracheal intubation. Learners will be able to able to optimize patient positioning, physiology, and equipment to safely intubate a premature neonate.

**Equipment or Props Needed:**

Premature infant manikin, infant warmer, plastic bag (gallon sized), monitors, 24 gauge peripheral IV, infant bag-valve mask (BVM), intubation supplies (Miller 00, Miller 0, Miller 1; Endotracheal Tubes (ETT) – 2.0, 2.5, 3.0 cuffed, 3.5 uncuffed; C-MAC video laryngoscope Mac 0, Miller 0; neonatal LMA, T-Piece Resuscitator, nasal cannula, flexible catheter suction, stethoscope, rapid sequence intubation (RSI) medications, epinephrine, moulage (cyanosis).

**Actors Needed:**

There are two facilitators. One can act as a respiratory therapist (RT), providing positive pressure ventilation (PPV) via NeoPuff. The other facilitator can obtain the equipment/medications requested by the trainee, provide history, and prompt them to proceed through the case, if needed.

**Stimulus Inventory:**

None

**Background and brief information:** A 0-day-old male was born at 31 weeks estimated gestational age to a G2P1 mother who delivered in her car at the freestanding ED entrance 5 minutes ago. The baby had a normal 20-week ultrasound. He weighs 1400 grams.

**Initial presentation:** The patient presents to you limp, cyanotic, and with a weak cry. He was immediately placed in the warmer in a bag, and stimulated, following the Neonatal Resuscitation Program (NRP) algorithm. Despite this, the baby was still gasping. The nurse obtained an IV, and the trainee should decide that it is time to intubate.

**How the scene unfolds:** The trainee should initiate PPV and go through the steps of MR SOPA (Mask readjustment, Reposition head, Suction, Open mouth, Positive pressure, Alternative airway). After reaching the “Positive pressure” step and increasing the pressure, the patient’s HR is 80 bpm and SpO2 is 78%. Trainees should verbalize intent to intubate without RSI medications. However, if they choose to intubate with RSI (incorrect choice), it will take 60 seconds to obtain the medications and 45 seconds for the medications to take effect; meanwhile, the patient will continue to desaturate and become bradycardic. If they start compressions, remind the trainee that NRP requires establishing an advanced airway prior to compressions. If the trainees accidentally esophageally intubate the neonate, the patient will also desaturate and become bradycardic.

**Critical actions:**

1. Start PPV (21–30% FiO2, PEEP 5, PIP 20)
2. MR SOPA steps (Max FiO2 100%, Max PIP 40, Max PEEP 10)
3. Successful intubation with appropriately sized intubation equipment and without RSI medications

**Chief Complaint:** A 0-day-old male was born at 31 weeks estimated gestational age to a G2P1 mother who delivered in her car at the freestanding ED entrance 5 minutes ago. The baby had a normal 20-week ultrasound. He weighs 1400 grams.

The patient was immediately placed in the warmer in a bag and stimulated, following the NRP algorithm. Despite this, the baby was still gasping. Your nurse obtained an IV and asks you if you want to intubate as you start to perform PPV.

**Table:**

**Vitals:**	Heart Rate (HR) 70	Blood Pressure (BP) --/--	Respiratory Rate (RR) 40 (PPV)
	Temperature (T) --	Oxygen Saturation (O_2_Sat) 75%	

**General Appearance:** Limp, cyanotic

**Primary Survey:**

- **Airway:** Occasional weak cry
- **Breathing:** Coarse breath sounds bilaterally, chest rise with PPV is limited
- **Circulation:** Sinus bradycardia, cyanotic, mottled

**History**:

**History of present illness:** A 0-day-old male was born at 31 weeks estimated gestational age to a G2P1 mother who delivered in her car at the freestanding ED entrance 5 minutes ago. He weighs 1400 grams.

- **Past medical history:** The baby had a normal 20-week ultrasound.
- **Past surgical history:** None
- **Patient’s medications:** None
- **Allergies:** None
- **Social history:** None
- **Family history:** None

**Secondary Survey/Physical Examination:**

- **General appearance:** Limp, cyanotic
- **HEENT:** Normal
- **Heart:** Bradycardic, no obvious murmurs
- **Lungs:** Coarse breath sounds bilaterally, limited chest rise with PPV
- **Abdominal/GI:** Normal
- **Genitourinary:** Normal
- **Rectal:** Normal
- **Extremities:** Cyanotic, mottled
- **Back:** Normal
- **Neuro:** Limp, moves all extremities when being stimulated and during IV start
- **Skin:** Cyanotic, mottled. Peripheral IV (PIV) in right foot
- **Lymph:** Normal
- **Psych:** Normal

**Results:**

- If asked, POC glucose is 60.

## OPERATOR MATERIALS

*Station 1: Premature Neonate Airway*

**Diagnosis:**

Respiratory failure secondary to RDS

**Disposition:**

NICU

## SIMULATION ASSESSMENT

*Station 1: Premature Neonate Airway*

Learner: _________________________________________

***Assessment Timeline***

This timeline is to help observers assess their learners. It allows observer to make notes on when learners performed various tasks, which can help guide debriefing discussion.

## DEBRIEFING AND EVALUATION PEARLS

*Station 1: Premature Neonate Airway*

**Pearls:**

- Neonatal airways have many anatomic and physiologic differences compared to airways of older children or adults 20–24
  ○ Bradycardia is primarily driven by hypoxia in this population
  ○ Neonates have different saturation goals based on minute and hour of life
  ○ Prior to considering intubation, corrective steps should be completed
- MR SOPA is an acronym that can be used for the ventilation corrective steps^22^,^24^
  ○ Mask Readjustment
  ○ Reposition Airway
  ○ Suction mouth and nose
  ○ Open mouth
  ○ Positive pressure
  ○ Alternative airway
- LMAs can be used in infants 2 kg or greater (approximately corresponding to a 34-week infant) 22,24
- Intubation is completed without RSI medications^22^,^25^
- Neonatal ETT size and blade size is based on gestational age and weight^22^
  ○ <28 weeks gestation: 2.5 endotracheal tube (ETT) and 00 or 0 blade
  ○ 28–34 weeks gestation 3.0 ETT and 0 blade
- Intubation attempts should be limited to 30–45 seconds in neonates due to low reserves^22^,^25^
- Lip lift will help to increase space in the oral cavity^22^,^24^

**Other debriefing points:**

- Why was this a difficult intubation?
- Review neonate airway anatomy (if it does not come up in the previous discussion) 20–23
  ○ Relatively large tongue, shorter neck, larynx is higher and more cone shaped, trachea is shorter/narrower/higher in the neck/more anterior, epiglottis that is narrower/larger/longer/and less flexible, cricoid cartilage is narrowest part of the airway (vs. adults where it is the glottic opening).
- Review premature neonate airway physiology (if it does not come up in the previous discussion).^20^–^24^
  ○ Low pulmonary compliance and high resistance – lungs are still filled with amniotic fluid, and premature neonates have decreased surfactant levels.
  ○ Positive pressure is key.
  ○ Low reserves – they desaturate quickly. Limit intubation attempts to 30–45 seconds.
  ○ Some early studies on high-flow nasal cannula (HFNC) for apneic oxygenation in non-emergent neonatal intubation (not this case, which is emergent).
  ○ Review targeted preductal SpO2 after birth (80–85% at 5 minutes, 85–95% at 10 minutes).
- Review when an advanced airway is needed:^22^,^24^
  ○ Usually, the primary cause of neonate bradycardia is hypoxia.
  ○ An advanced airway is indicated when PPV/bag-valve mask (BVM) and MR SOPA troubleshooting does not improve oxygenation, or you do not have chest wall movement despite MR SOPA.
  ○ Remember NRP target pre-ductal oxygen saturation after birth.
  ○ No compressions until an advanced airway (ETT or LMA – if > 2 kg – is established).
  ○ What is the lowest weight infant you can use an LMA in? 2 kg (~34 weeks, could be down to 32–33).
  ○ When might you need a Miller 00 blade?
  ○ What size ETT and blade for a 1–2 kg (28–32 week) neonate? 22
- What are some challenges you anticipate with this intubation?^22^
  ○ Lots of secretions – have suction ready.
  ○ “Anterior” airway.
  ○ Neck over- or under-extension. Under-extension: will not be able to reach the cords. Over-extension: will not be able to pass the ETT.
  ○ Not able to pass the tube: backward upward rightward pressure (BURP maneuver) or cricoid pressure. Can also rotate the ETT.
  ○ Head rolling – make sure to stabilize the head with your right hand.
    ▪ The C-MAC/video blade takes up a lot of space in the oral cavity – can have an assistant do a right-sided lip pull/lift to give you space to pass the tube. You may also need to switch to a DL blade to give yourself more room.
    ▪ No RSI – different from what we are used to. The cords may be closed, may be harder to control tongue.
- If this were a nonemergent neonatal intubation (example, a 30-weeker delivered at home comes in several hours later with respiratory distress syndrome and does not improve with CPAP), RSI is appropriate: atropine + etomidate/fentanyl/versed + paralytic. Depending on your local guidelines, no ketamine under 1–3 months.^22^,^25^
- Time for practice!
  ○ Practice looking into the oropharynx with a Mac 0 vs Miller 0 CMAC blades
  ○ Practice looking into the oropharynx with a Mac 0 vs Miller 0 DL blades
  ○ Best techniques for head stabilization and positioning for neck under/over extension
  ○ Practice BURP/cricoid and rotating the ETT
  ○ Practice 2-person PPV, including verifying appropriate size mask, 2-person technique for jaw thrust/head tilt.

**Brief Wrap-up:**

- Assist the learners in looking at the overall experience.
- Time permitting, ask the learners to share their top take-home points, and clarify how the learners’ take-home points will transfer to their clinical practice.
- If time is limited, the facilitator can give a quick summary of the main take-home points.
- Link the learning back to simulation goals and objectives.
- Thank the learners for their participation and feedback.

## INSTRUCTOR MATERIALS

*Station 2: Obese Patient*

**Case Title:** Obese Patient

**Case Description & Diagnosis (short synopsis):** An obese 17-year-old male was found unresponsive at home and ultimately needed endotracheal intubation. Trainees will learn how to optimize patient positioning and preoxygenation to safely intubate obese patients.

**Equipment or Props Needed:**

Obese manikin (or regular adult-sized manikin with pillow padding on back and front to simulate obese body habitus), monitors, IV, BVM, RSI medications, intubation supplies (Mac 3 and 4; 7.0, 7.5, 8.0 cuffed ETT; C-MAC Mac 3, 4, and/or adult hyperangulated blade; bougie), Yankauer suction, LMA, non-rebreather (NRB) mask, nasal cannula (NC), high flow nasal cannula (HFNC), blankets or pillows for ramping patient, moulage (vomitus on manikin face).

**Actors Needed:**

The facilitator may act as a bedside nurse

**Stimulus Inventory:**

**Background and brief information:** Kevin is a 17-year-old obese male who was found unresponsive at home by his parents with an empty bottle of Seroquel (Quetiapine 300 mg XR) next to him, and brought to you at the free-standing ED. Glucose was reportedly normal for EMS, and they have placed an IV in the L antecubital (AC) fossa. Immediately upon arrival, the patient vomits, and you suction and decide to intubate.

**Initial presentation:** Kevin initially presents to your ED in an obtunded state; he will react to noxious stimuli only and is not protecting his airway.

**How the scene unfolds:** The trainee should immediately recognize that an advanced airway is needed and should verbalize the intent to intubate. The trainee should also recognize the patient’s obesity as a challenge to successful intubation. The patient should be preoxygenated and properly placed in a ramped position. If the trainee does not adequately preoxygenate or ramp the patient prior to intubation attempt, the patient will become more hypoxic, go into pulseless electrical activity (PEA) arrest, and the scenario will end.

**Critical actions:**

1. Preoxygenate the patient while preparing for intubation.
2. Ramp the patient to optimize positioning prior to intubation.

**Chief Complaint:** A 17-year-old obese male was found unresponsive at home after a medication overdose.

**General Appearance:** Somnolent, responds to pain only. Vomit on face. Obese.

**Primary Survey:**

- **Airway:** Normal
- **Breathing:** Shallow breaths, clear lungs bilaterally
- **Circulation:** Normal

**History:**

- **History of present illness:** Kevin is an obese 17-year-old male who was found unresponsive at home by his parents with an empty bottle of Seroquel (Quetiapine 300 mg XR) next to him, and brought to you at the free-standing ED. Glucose was normal, and they have placed an IV in the L antecubital (AC) fossa. Immediately upon arrival, the patient vomits and you suction and decide to intubate. Please choose your intubation equipment and tell me your RSI meds and how you would like to preoxygenate the patient.
- **Past medical history:** Obstructive sleep apnea (OSA)
- **Past surgical history:** None
- **Patient’s medications:** None
- **Allergies:** None
- **Social history:** None
- **Family history:** None

**Secondary Survey/Physical Examination:**

- **General appearance:** Somnolent, responds to pain only. Vomit on face. Obese.
- **HEENT:** Normal
- **Neck:** Normal
- **Heart:** Normal
- **Lungs:** Shallow breaths, clear lungs bilaterally
- **Abdominal/GI:** Normal
- **Genitourinary:** Normal
- **Rectal:** Normal
- **Extremities:** Normal
- **Back:** Normal
- **Neuro:** Pupils 5 mm, sluggish, Glascow Coma Scale (GCS) is 7 (eyes 1, verbal 2, motor 4), incomprehensible moaning, withdraws to pain
- **Skin:** PIV in left arm
- **Lymph:** Normal
- **Psych:** Normal

**Results:**

- If asked, glucose 130, and no QTc prolongation on the monitor

## OPERATOR MATERIALS

*Station 2: Obese Patient*

**Diagnosis:**

Respiratory failure secondary to medication overdose.

**Disposition:**

PICU

## SIMULATION ASSESSMENT

*Station 2: Obese Patient*

Learner: _________________________________________

***Assessment Timeline***

This timeline is to help observers assess their learners. It allows observer to make notes on when learners performed various tasks, which can help guide debriefing discussion.

## DEBRIEFING AND EVALUATION PEARLS

*Station 2: Obese Patient*

**Pearls:**

- Patients with body mass index (BMI) >35 have anatomic and physiologic differences and are likely to desaturate rapidly^26^–^28^
- Optimal pre-oxygenation is in the upright or semi-upright position^28^–^30^
- If possible, consider pre-oxygenation with high flow nasal cannula (HFNC) or bilevel positive airway pressure (BiPAP)^29^,^30^
- Optimal head positioning is in ear-to-sternal notch position^28^–^30^
- RSI dosing is controversial, but consider using ideal body weight (IBW) for initial medication dosing^26^–^28^
- Consider use of a hyperregulated blade for improved glottic views^27^,^28^
- Tidal volume should be based on IBW not total body weight (TBW)^26^–^28^

**Other debriefing points:**

- If the patient had significant hypoxia or coded during the first run-through:^26^–^28^
  ○ Why do you think this patient desaturated so rapidly?
    ▪ Anatomic differences in the patient with BMI >35: Increased airway resistance, elevated diaphragm, increased chest wall resistance, lower functional reserve capacity, larger tongue, increased chance of pharyngeal wall collapse with lying flat and with RSI.
    ▪ Due to increased mass of the chest wall, as well as high prevalence of OSA, obese patients will have optimal oxygenation and ventilation in the upright or semi-upright position.^29^,^30^
    ▪ May have worse glottic view - may benefit from a bougie or a D-blade. 27,28
    ▪ Physiologic differences:
      - Increased O2 consumption and carbon dioxide (CO2) production-> lower safe apnea time.
      - RSI medication dosing in obese patients is very controversial, and almost no pediatric data to guide medication dosing.
      - If you dose opioids, benzodiazepines, or propofol based on TBW in patients with BMI >40, you can cause respiratory depression, apnea, and hypotension.^31^,^32^ For your standard ketamine/rocuronium, dose based on ideal (or lean or adjusted, this is controversial) BW not TBW – for smaller children, you can use the Broselow tape for a quick BW.

If the patient did not desaturate the first time:

- Why did you ramp the patient, use HFNC/apneic oxygenation?
- Discuss same points as above.

What can we do to prevent rapid desaturation?^28^–^30^

- Ramp patient in the ear to sternal notch position – should do this for BMI > 35
- How do you ramp?^29^
- Make sure you’re not ramping incorrectly! Do not hyperextend the neck.
- If ramping not possible or BMI <35, keep the patient semi-upright/semi-Fowler’s if possible to improve oxygenation and ventilation.
- Consider (if urgent, but not emergent intubation, and at an ED with operating room (OR)/otolaryngology (ENT)/anesthesia in house) calling for anesthesia/OR intubation for awake intubation +/− fiberoptic intubation.
- If your group already knows everything about ramping and oxygenating for this case, can potentially discuss and try upright intubation, practice with bougie or D-blade, or discuss non-fiberoptic options for awake intubation.
- How can you optimize BVM in this patient?
  ○ Perform 2-handed mask seal.
  ○ Discuss pre-oxygenation options: NC at 15 liters per minute (LPM) (or 5 LPM for infants-toddlers, 10 LPM for school age, 15 LPM for teenagers) + NRB at flush or HFNC 2L/kg to max 60 L/min. 26–28
- Discuss apneic oxygenation. HFNC is great since you can use it for both, but in this case, would not have been ready in time.
- When might you preoxygenate with continuous positive airway pressure (CPAP)/Bi-level positive airway pressure (BiPAP)? Why might you not do that in this case?
- Can mention that for some patients, delayed sequence intubation (DSI) with ketamine + BiPAP or ketamine + HFNC is an option. In this actively vomiting patient, BiPAP is not an option.
- How do we adjust tidal volume for ventilator settings? Based on IBW/height, not TBW.^26^

After your brief debrief, run the scenario again!

**Brief Wrap-up:**

- Assist the learners in looking at the overall experience.
- Time permitting, ask the learners to share their top take-homes, and clarify how the learners’ take-homes will transfer to their clinical practice.
- If time is limited, the facilitator can give a quick summary of the main take-homes.
- Link the learning back to simulation goals and objectives.
- Thank the learners for their participation and feedback.

## INSTRUCTOR MATERIALS

*Station 3: Inhalational Injury*

**Case Title:** Inhalational Injury

**Case Description & Diagnosis (short synopsis):** A 3-year-old patient was rescued from a house fire with respiratory distress and ultimately needed endotracheal intubation. Learners will be able to manage the airway of a patient with an inhalational injury by recognizing impending respiratory failure and successfully intubating the patient.

**Equipment or Props Needed:**

Pediatric burn manikin, nasal cannula (NC), non-rebreather mask (NRB), monitors, peripheral IV (PIV) supplies, bag-valve mask (BVM), IV fluids, RSI medications, inhalational racemic epinephrine, albuterol, aerosol device, Yankauer suction, intubation supplies (Mac 2, Miller 2; 3.0, 3.5, 4.0, 4.5 cuffed ETT; LMA; Video Laryngoscope: Mac 2, pediatric hyperangulated blade), moulage (soot, burns).

**Actors Needed:**

The facilitator may act as a bedside nurse

**Stimulus Inventory:**

**Background and brief information:** 3-year-old previously healthy male who was found at the scene of a house fire just prior to arrival at your free-standing ED. SpO2 92% on room air, EMS has started racemic epinephrine via nebulizer and have established an IV. Racemic epinephrine finishes as EMS arrives and they take the mask off for transfer to your stretcher.

**Initial presentation:** The patient is awake and alert, but in respiratory distress and stridulous. He appears panicked and he has soot in his nares.

**How the scene unfolds:** The trainees should quickly recognize that the patient is in respiratory distress and stridulous. They should apply supplemental O2 to preoxygenate as they prepare for intubation to protect the patient’s airway. If the trainees have improper preoxygenation, delay intubation, have a prolonged intubation attempt, or use improper intubation supplies, the patient becomes profoundly hypoxic, arrests, and the scenario ends.

**Critical actions:**

1. Immediate preoxygenation and recognition that the patient will require an advanced airway.
2. Intubation with appropriately sized airway equipment, considering that the airway may be more edematous.
3. Utilize LMA quickly as a temporizing airway if needed.

**Chief Complaint:** 3-year-old male who presents from a house fire in respiratory distress.

**General Appearance:** Crying with a hoarse voice, in distress

**Primary Survey:**

- **Airway:** Soot in mouth, thick secretions
- **Breathing:** Tachypneic, scattered end-expiratory wheeze, inspiratory stridor
- **Circulation:** Tachycardic

**History:**

- **History of present illness:** 3-year-old previously healthy male who was found at the scene of a house fire just prior to arrival at your free-standing ED. SpO2 92% on room air, EMS has started giving racemic epinephrine via nebulizer and have established an IV. Racemic epinephrine finishes as EMS arrives and they take the mask off for transfer to your stretcher.
- **Past medical history:** Unknown
- **Past surgical history:** Unknown
- **Patient’s medications:** Unknown
- **Allergies:** Unknown
- **Social history:** Unknown
- **Family history:** Unknown

**Secondary Survey/Physical Examination:**

- **General appearance:** Crying with a hoarse voice, in distress
- **HEENT:**
  ○ **Head:** Normal
  ○ **Eyes:** Normal
  ○ **Ears:** Normal
  ○ **Nose:** Soot in bilateral nares
  ○ **Throat:** Soot in mouth, thick secretions
- **Neck:** Normal
- **Heart:** Normal
- **Lungs:** Tachypneic, scattered end-expiratory wheeze, inspiratory stridor
- **Abdominal/GI:** Normal
- **Genitourinary:** Normal
- **Rectal:** Normal
- **Extremities:** Normal
- **Back:** Normal
- **Neuro:** Agitated
- **Skin:** Soot on face, burns to hands. PIV in left arm
- **Lymph:** Normal
- **Psych:** Normal

**Results:**

- If asked, glucose 120

## OPERATOR MATERIALS

*Station 3: Inhalational Injury*

**Diagnosis:**

Acute hypoxic respiratory failure secondary to inhalational burn injury.

**Disposition:**

PICU

## SIMULATION ASSESSMENT

*Station 3: Inhalational Injury*

Learner: _________________________________________

***Assessment Timeline***

This timeline is to help observers assess their learners. It allows observer to make notes on when learners performed various tasks, which can help guide debriefing discussion.

## DEBRIEFING AND EVALUATION PEARLS

*Station 3: Inhalational Injury*

**Pearls:**

- Inhalational injuries are often from exposure to thermal injury. Patients who are trapped in enclosed spaces (eg, house fire requiring extrication) are at higher risk. 33–35
- Upper airway injury signs: discoloration, soot in airway, stridor, hoarse voice. 33–35
- Lower airway injury signs: coughing, wheezing or dyspnea. 33–35
- Progressive airway injury occurs over 24–48 hours. 33–35
- Early intubation for patients at high risk for progressive injury is the mainstay treatment for inhalational injuries. 33–35
- In the stable patient, can consider bedside scope to evaluate extent of inhalational injury prior to determining airway management strategy. 33–35
- Consider racemic epinephrine and/or albuterol while preparing for intubation. 33–35
- Consider pre-oxygenation with HFNC if possible. 33–35
- Presume carbon monoxide poisoning until excluded and treat with 100% oxygen. 33–35
- When considering ETT size, it is good practice to have ETT of estimated size and up to 2 sizes smaller in the case of extensive airway edema. 33–35
- Consider calling the difficult airway team if available. 33–35
- Back-up airway will be supraglottic airway, but be prepared for a surgical airway.^33^–^35^

**Other debriefing points:**

- How did that go?
- What went well?
- What would you do differently?
- If there is time, let learners go back to the manikin to try to get a good view of the cords.
- Review inhalation injury pathophysiology (can keep the pathophysiology to a minimum if there are time constraints):^33^–^35^
  ○ Upper airway/laryngeal inhalation injury: often due to thermal injury. Leads to rapid development of edema. Can cause significant distortion of oral and laryngeal anatomy. If combined with smoke inhalation, there may also be significant discoloration. May manifest as hoarse voice or stridor.
  ○ Lower airway inhalation injury: due to inhaled smoke. Leads to inflammation, edema, and mucosal sloughing. May manifest as coughing, wheezing, dyspnea.
  ○ Inhalation injuries are one of the leading causes of death in burn victims (20–30% mortality in pediatric patients).
  ○ It is important to know the circumstances of the burn injury – enclosed vs. non-enclosed. Patients trapped in enclosed spaces such as house fires and car fires with prolonged extrication are at greatest risk for inhalation injury.
  ○ Early recognition is key in pediatric patients – due to the smaller diameter of their trachea, even small amounts of edema can cause severe respiratory distress and difficulty with intubation/passing the endotracheal tube.
  ○ Injury progresses over the first 24–48 hours. Patients may present with minimal symptoms. Early intubation has been a mainstay treatment for inhalation injuries because delay may lead to a very difficult intubation due to worsening edema as well as worsening physiology (pulmonary injury, shock, and infection).
- When do you intubate a patient with inhalational injury?^33^–^35^
  ○ Some criteria are not controversial: stridor, respiratory distress, edema on nasopharyngoscopy/laryngoscopy, intraoral burns, altered mental status, failure to oxygenate or ventilate, large/severe burn with uncontrolled pain, full thickness facial burns, upper airway trauma, and hemodynamic instability. Suspected smoke inhalation is often also included.
  ○ Traditionally, the following conservative criteria were also indications for intubation: facial or oropharyngeal soot, hoarseness, dysphagia, singed facial hair, or any facial burns.
  ○ Retrospective studies have shown that as many as 30% of inhalation injury patients may be intubated unnecessarily.^36^
  ○ Hoarseness and dysphagia are controversial.
  ○ If the patient is stable and able to cooperate, ENT can scope at bedside to evaluate the extent of inhalation injury.
  ○ In pediatric patients, err on the side of caution. Pediatric patients have limited reserve, smaller tracheas, and may not be able to cooperate with scoping at bedside.
  ○ Can also discuss how the need to transport the patient affects the decision to intubate.
- Intubation pearls:^33^–^35^
  ○ If patient is semi-stable, consider adjuncts including racemic epinephrine or albuterol prior to intubation.
  ○ Consider using HFNC for pre-oxygenation and apneic oxygenation, if readily available.
  ○ Assume laryngeal swelling and have ETT up to 2 sizes smaller available.
  ○ Expect edema of the tongue, epiglottis, and arytenoids.
  ○ Excessive fluid resuscitation can exacerbate laryngeal swelling, increasing the difficulty of tracheal intubation.
  ○ A bougie or pediatric D-blade may be helpful to navigate edema and poor laryngoscopic view.
  ○ LMA is still your initial backup but may not have a good seal due to edema.
  ○ Consider calling anesthesia for airway backup.
  ○ Awake look and awake fiberoptic intubation can be considered
    ▪ When might you do an awake look?
  ○ May need special ETT securement if there are facial burns (can use an ETT holder, or ties/umbilical tape).
  ○ BVM, mouth opening, and neck extension may be difficult if there are facial burns. The need for a surgical airway is always a possibility in burn patients.
- Other adjuncts/considerations to help manage the airway?^35^
  ○ Humidified oxygen helps to avoid the development of thickened secretions.
  ○ Bronchodilators that are useful in the treatment of inhalation injury include albuterol for wheezing/bronchospasm, and racemic epinephrine for stridor or retractions. EMS’s choice of racemic epinephrine prehospital was a good one!
  ○ Carbon monoxide poisoning should be presumed in any patient who presents following smoke inhalation until it is excluded by a normal carboxyhemoglobin level. Place all non-trivial burn patients on 100% oxygen via NRB.

**Brief Wrap-up:**

- Assist the learners in looking at the overall experience.
- Time permitting, ask the learners to share their top take-homes, and clarify how the learners’ take-homes will transfer to their clinical practice.
- If time is limited, the facilitator can give a quick summary of the main take-homes.
- Link the learning back to simulation goals and objectives.
- Thank the learners for their participation and feedback.

## INSTRUCTOR MATERIALS

*Station 4: Contaminated Airway*

**Case Title:** Contaminated Airway

**Case Description & Diagnosis (short synopsis):** 16-year-old female who presents with altered mental status and emesis, who required intubation. Learners are to understand strategies and demonstrate techniques to safely intubate the patient with a contaminated airway.

**Equipment or Props Needed:**

Emesis manikin (Sampson C, Pauly J, Horner J, 2018), monitors, moulage (emesis to face), bag-valve mask (BVM), intubation supplies (Mac 3 and 4; 7.0, 7.5, 8.0 cuffed ETT; Video laryngoscope: Mac 3, 4), Yankauer suction x2, laryngeal mask airway (LMA), oropharyngeal airway (OPA), non-rebreather mask (NRB), peripheral IV (PIV), RSI medications.

**Actors Needed:**

The facilitator may act as a bedside nurse

**Stimulus Inventory:**

**Background and brief information:** Maria is a 16-year-old who presents with sudden onset of headache and altered mental status at school. As she came through the ambulance bay doors, she had a brief tonic-clonic seizure, now stopped, followed by a large volume emesis. You decide to intubate and have completed preoxygenation with a NRB; your nurse is about to push RSI meds through an established IV.

**Initial presentation:** Maria initially presents in an obtunded state with labored breathing and emesis in her mouth.

**How the scene unfolds:** The trainee should initially address the contaminated airway by requesting suction. The trainee should also continue to preoxygenate and request intubation supplies. The facilitator is to push RSI medications at 90 seconds and force the trainee to intubate with a contaminated airway. If no successful intubation within 30 seconds, the patient becomes profoundly hypoxic, arrests, and the scenario ends.

**Critical actions:**

1. Have multiple suction devices prepared (Yankauer x2) when intubating contaminated airways.
2. Use appropriate suctioning technique to successfully intubate the patient with large volume emesis.

**Chief Complaint:** A 16-year-old with altered mental status who presents after a brief tonic-clonic seizure followed by a large volume emesis. She is not protecting her airway.

**General Appearance:** Minimally responsive, face covered in emesis.

**Primary Survey:**

- **Airway:** Contaminated with copious amounts of vomitus
- **Breathing:** Labored, lungs coarse bilaterally
- **Circulation:** Normal

**History:**

- **History of present illness:** Maria is a 16-year-old who presents with sudden onset of headache and altered mental status at school. As she came through the ambulance bay doors, she had a brief tonic-clonic seizure, now stopped, followed by a large volume emesis.
- **Past medical history:** Unknown
- **Past surgical history:** Unknown
- **Patient’s medications:** Unknown
- **Allergies:** Unknown
- **Social history:** Unknown
- **Family history:** Unknown

**Secondary Survey/Physical Examination:**

- **General appearance:** Minimally responsive, face covered in emesis
- **HEENT:**
  ○ **Mouth:** Contaminated with copious amounts of vomitus
- **Neck:** Normal
- **Heart:** Normal
- **Lungs:** Coarse breath sounds bilaterally
- **Abdominal/GI:** Normal
- **Genitourinary:** Normal
- **Rectal:** Normal
- **Extremities:** Normal
- **Back:** Normal
- **Neuro:** Pupils 3 mm, sluggish, Glascow Coma Scale (GCS) is 7 (eyes 1, verbal 2, motor 4), incomprehensible moaning, withdraws to pain
- **Skin:** Vomitus on face. PIV in left arm.
- **Lymph:** Normal
- **Psych:** Normal

**Results:**

- If asked, POC glucose is 120

## OPERATOR MATERIALS

*Station 4: Contaminated Airway*

**Diagnosis:**

Respiratory failure secondary altered mental status, complicated by a contaminated airway.

**Disposition:**

PICU

## SIMULATION ASSESSMENT

*Station 4: Contaminated Airway*

Learner: _________________________________________

***Assessment Timeline***

This timeline is to help observers assess their learners. It allows observer to make notes on when learners performed various tasks, which can help guide debriefing discussion.

## DEBRIEFING AND EVALUATION PEARLS

*Station 4: Contaminated Airway*

**Pearls:**

- Airways can be contaminated by various substances such as emesis, pulmonary edema, secretions, or blood, which can make visualization difficult.^23^,^33^,^37^
- Suctioning optimization will help with intubation success.^39^
  ○ Double suction
  ○ Large bore suction
  ○ Using ETT as suction
  ○ Suction assisted laryngoscopy and airway decontamination (SALAD) technique.
- Consider airway adjuncts, such as use of a bougie, to optimize success.^23^,^33^,^37^,^38^
- Supraglottic airway or bag-mask ventilation may not be successful if significant contamination.^23^,^33^,^37^,^38^

**Other debriefing points:**

- How did that go**?**
- What made it so difficult**?**
- What are common causes of the contaminated airway**?** 23,33,37
- Pulmonary edema
  ○ Emesis/regurgitation
  ○ Excess oral secretions (ketamine can cause this)
  ○ Blood (traumatic, atraumatic)
  ○ Some combination of the above
- What are the dangers of the contaminated airway**?**^23^,^33^,^37^,^38^
  ○ Difficult anatomically – can**’**t visualize landmarks.
    ▪ May have edema of laryngeal structures.
  ○ Technically not physiologically difficult. But if you do not secure the airway, it can lead to desaturation.
  ○ Can lead to aspiration, difficulty with ventilation post-intubation.
  ○ Is an LMA a viable backup option**?**
  ○ Is BVM a viable backup option**?**
  ○ What is your backup option**?**
- How can you optimize suction**?**^39^
  ○ Yankauer optimization
  ○ Double suction setup
  ○ Large bore suction (eg, DuCanto)
  ○ SALAD (suction assisted laryngoscopy airway decontamination)
  ○ Suctioning through large bore ETT
- How else can you troubleshoot this intubation**?**^39^
  ○ Intentionally intubating the esophagus and either inflate the balloon and/or connect the ETT to suction, bougie, surgical airway.
- The scenario as written will take a maximum of 3 minutes. Immediately after debriefing and discussion, go back and let each group member intubate with double suction setup.

**Brief Wrap-up:**

- Assist the learners in looking at the overall experience.
- Time permitting, ask the learners to share their top take-home points, and clarify how the learners**’** take-home points will transfer to their clinical practice.
- If time is limited, the facilitator can give a quick summary of the main take-home points.
- Link the learning back to simulation goals and objectives.
- Thank the learners for their participation and feedback.

## INSTRUCTOR MATERIALS

*Station 5: Failed Airway*

**Case Title:** Failed Airway

**Case Description & Diagnosis (short synopsis):** A 1-year-old male who presents with respiratory failure secondary to a seizure, and he needed endotracheal intubation. The manikin settings will be set to make it likely that the learners will fail their first intubation attempt. Learners will recognize when an intubation attempt has become a **“**failed airway**”** and use airway adjuncts to rescue their patient.

**Equipment or Props Needed:**

Infant manikin with inflatable tongue (set to maximum possible edema) or pediatric difficult airway trainer with option for tongue swelling, bag-valve mask (BVM), monitors, peripheral IV (PIV), RSI medications, intubation supplies (Miller 1, Video laryngoscope: Miller 1 and Miller 2, pediatric hyperangulated blade, ETT 3.5, 4.0, pediatric bougie), nasopharyngeal airway (NPA), oropharyngeal airway (OPA), laryngeal mask airway (LMA), moulage (blood around mouth).

**Actors Needed:**

The facilitator may act as a bedside nurse

**Stimulus Inventory:**

**Background and brief information:** 1-year-old male presents to the freestanding ED with seizures. This morning while he was playing, he started **“**shaking all over.**”** Mom called EMS who gave him intramuscular (IM) midazolam once, which stopped the seizure. They have established an IV en route. On arrival to the ED, the patient is responsive to pain only, with equal but sluggishly reactive pupils.

**Initial presentation:** On arrival to the ED, the patient is responsive to pain only, with equal but sluggishly reactive pupils. He develops apnea. RT starts bagging, and you make the decision to intubate. You have RSI medications ready, and your nurse is ready to push them.

**How the scene unfolds:** Trainees will attempt intubation, only to fail with subsequent hypoxia, and to thus have a failed airway. They will need to utilize airway adjuncts, such as an OPA or LMA, to keep their patient stable before their second intubation attempt.

**Critical actions:**

1. Recognize a failed airway
2. Be able to utilize airway adjuncts in a failed airway scenario

**Chief Complaint:** 1-year-old male who is post-ictal and unable to protect his airway. He has blood around his mouth.

**General Appearance:** Limp, unresponsive

**Primary Survey:**

- **Airway:** Small amount of blood around the mouth. Tongue large and edematous.
- **Breathing:** Coarse breath sounds bilaterally, being bagged with BVM.
- **Circulation:** Flushed skin

**History:**

**History of present illness:** 1 year-old male presents to the freestanding ED with seizures. This morning while he was playing, he started shaking. Mom called EMS who gave him IM midazolam once, which stopped the seizure. They have established an IV en route. He is febrile.

**Past medical history:** Dad with seizures that he **“**grew out of.**”**

- **Past surgical history:** None
- **Patient's medications:** None
- **Allergies:** None
- **Social history:** None
- **Family history:** None

**Secondary Survey/Physical Examination:**

- **General appearance:** Limp and unresponsive
- **HEENT:**
  ○ **Head:** Normal
  ○ **Eyes:** Normal
  ○ **Ears:** Normal
  ○ **Nose:** Soot in bilateral nares
  ○ **Throat:** Small amount of blood around the mouth. Tongue large and edematous.
- **Neck:** Normal
- **Heart:** Normal
- **Lungs:** Normal
- **Abdominal/GI:** Normal
- **Genitourinary:** Normal
- **Rectal:** Normal
- **Extremities:** Normal
- **Back:** Normal
- **Neuro:** Obtunded
- **Skin:** PIV in left arm
- **Lymph:** Normal
- **Psych:** Normal

**Results:**

- If asked, POC glucose is 120

## OPERATOR MATERIALS

*Station 5: Failed Airway*

**Diagnosis:**

Respiratory failure secondary to seizures complicated by a failed airway due to intraoral trauma.

**Disposition:**

PICU

## SIMULATION ASSESSMENT

*Station 5: Failed Airway*

Learner: _________________________________________

***Assessment Timeline***

This timeline is to help observers assess their learners. It allows observer to make notes on when learners performed various tasks, which can help guide debriefing discussion.

## DEBRIEFING AND EVALUATION PEARLS

*Station 5: Failed Airway*

**Pearls:**

**Pediatric airway adjuncts**

- Failed airway: Failure to maintain oxygenation or ≥ 3 attempts by an experienced operator. 33,41,42
- Adjunct options: oropharyngeal airway, supraglottic airway, bougie, hyperangulated blade.^23^,^33^,^41^,^43^
- The Storz hyperangulated pediatric blade is typically too large for neonatal/infant airways.^1^,^21^,^42^,^44^–^48^
- 4.0 ETT is the smallest that can fit a pediatric bougie.^40^

**Other debriefing points:**

- How did that go**?** What did you see when you looked in the mouth**?**
  ○ Review Cormack-Lehane grades.^2^,^3^,^40^
- Why was this a difficult airway**?**
- When did this become a failed airway (if it did)**?** If they got the intubation on the first attempt, you can ask, **“**What would you have done if you did not get the intubation on the first attempt and the patient**’**s oxygen saturation dropped**?”**
- What is the definition of a failed airway**?** Failure to maintain oxygenation or ≥ 3 attempts by an experienced operator.^33^,^41^,^42^
- What are your immediate next steps when you recognize a failed airway**?**^23^,^33^,^41^,^43^
  ○ If due to failure to oxygenate, bag immediately, ideally with an OPA. Have an LMA out if you anticipate a difficult intubation. If an extra-glottic device is unsuccessful or extremely unlikely to be unsuccessful (severe upper airway trauma, secretions, or epiglottis edema), needle cricothyrotomy is the next step.
- Now go back to practice with the airway adjuncts. The goal here is to give them as much time as possible to practice using airway adjuncts/difficult airway tools:^33^,^38^,^43^,^44^
  ○ LMA
  ○ OPA
  ○ Bougie
  ○ CMAC D-blade (or other hyperangulated laryngoscope blade)
- The D-blade is too large for the infant manikin but can be used with the pediatric airway trainer that should also be in the room.
- What is the smallest ETT you can fit over a pediatric bougie**?** 4.0^40^
- How is a hyperangulated D-blade different from a standard geometry blade**?**^1^,^21^,^42^,^44^–^48^
- What situations would a D-blade be helpful for**?**
  ○ Tongue edema
  ○ Limited mouth opening
  ○ Limited neck mobility

**Brief Wrap-up:**

- Assist the learners in looking at the overall experience.
- Time permitting, ask the learners to share their top take-home points, and clarify how the learners**’** take-home points will transfer to their clinical practice.
- If time is limited, the facilitator can give a quick summary of the main take-home points.
- Link the learning back to simulation goals and objectives.
- Thank the learners for their participation and feedback

## INSTRUCTOR MATERIALS

*Station 6: Physiologically Difficult Intubation*

**General pearls:**

- Three main buckets for the physiologically difficult airway: shock, hypoxia, and metabolic acidosis. There can be significant overlap.^45^
- Mnemonic = HOP Killers (Hypotension, Oxygenation, pH). 54
- Predictors of peri-arrest intubation in pediatric intubation: hypotension, myocardial dysfunction, persistent hypoxemia, severe metabolic acidosis (pH < 7.1), post-return of spontaneous circulation (ROSC), and status asthmaticus. 45, 51
- Most pure respiratory acidosis patients are not a physiologically difficult intubation (for example, intubating an apneic post-ictal patient) because the ventilation is the problem, and you can start fixing it with PPV immediately and fix it more long-term with an ETT. In metabolic acidosis, ventilation is your compensation, not the problem, and you stop your compensation when you intubate. PPV will not help much if you are already at maximum minute ventilation compensating for a metabolic acidosis. Pure respiratory acidosis is also unlikely to lead to very profound acidosis (pH < 6.9). 45,47
- Resuscitate before you intubate. You are rarely forced to act immediately. 45,47, 51
- If this patient comes to a community ED, consider calling transport as soon as possible to get to tertiary care center. For these stations, there is generally no one right answer. But there is a wrong answer, which is RSI before resuscitation/optimization. 42
- If a patient**’**s anticipated course is deterioration, often the sooner you can intubate after appropriate resuscitation, the better. 42

**Case 1: Refractory Hypoxia**

Your patient is a 3-month-old with bronchiolitis who has failed BiPAP despite sedation. The infant currently has a respiratory rate of 84 breaths per minute and an SpO2 of 90% on 100% FiO2. HR is 150 beats per minute, normal capillary refill and blood pressure. You decide to intubate.

What are you concerned will happen when you intubate this patient**?**

How can you optimize this patient prior to and during intubation**?**

**Learning points:**

- Patients who are hypoxic on 100% FiO2 (via HFNC or CPAP/BiPAP) have very low reserve. 47
- This patient is at high risk for rapid desaturation. This can lead to bradycardia, followed by cardiac arrest. 42
- This patient**’**s young age puts him at higher risk for rapid desaturation; infants and young children have low functional reserve capacity (FRC) but high oxygen consumption and high metabolic rates. 33
- Preoxygenation: optimizing technique for nitrogen washout and replacement by oxygen. 47
- Optimal preoxygenation/apneic oxygenation: 41,45,47
  ○ If this patient requires BiPAP (was much worse on HFNC), options include:
    ▪ Consider low-flow NC at bedside for apneic oxygenation. 5–15 LPM. One RT can pop it in as the other pulls off the mask. This can take under 5 seconds while you are opening the mouth and inserting the blade.
    ▪ If you still have the HFNC setup, you can keep the BiPAP mask on during RSI, and then as you go to intubate, RT can add HFNC on as the BiPAP mask comes off for apneic oxygenation during the intubation.
    ▪ If the patient is obtunded/sedated, you can consider preoxygenation with at least 8 tidal volume breaths with a BVM, with NC for apneic oxygenation during intubation.
  ○ If the patient was not completely failing HFNC (not hypoxic, but still with tachypnea/increased work of breathing or apnea), you can change back to HFNC for preoxygenation and leave it on for apneic oxygenation.
- Apneic oxygenation: All the ED/PICU studies for apneic oxygenation in children are unfortunately too variable to draw strong conclusions from. OR-based studies do show significant decrease in desaturation events and increased safe apnea duration for patients with apneic oxygenation. Again, no ED evidence, but we recommend using apneic oxygenation in all high-risk intubations. For apneic oxygenation, OR-based studies use 5 LPM for infants, 10 LPM for ages 1–8 years-old, and 15 LPM for 8+ years-old. You will not harm anyone by just doing 15 LPM for everyone. 43,46,47
- Can position patient with head of bed up to optimize oxygenation. 45,47
- Attempt for a low safe apnea time by having the most experienced intubator, or experienced intubator who is likely to be successful and complete the intubation in under 30 seconds, try for intubation first.^45^,^48^
- Anticipate post-intubation hypoxia. It may take time and PEEP/PIP to re-recruit this patient**’**s lungs after RSI.^47^,^48^
- Atropine: Pedatric Advanced Life Support no longer recommends routine use; however, in a patient likely to desaturate during RSI, atropine may help you avoid bradycardia and arrest. Atropine, however, will not fix your hypoxia. Decrease your hypoxia by the optimization steps above. 47,48, 50
- Rocuronium vs. succinylcholine – dose rocuronium at 1.2 mg/kg to minimize time to onset (mean onset of action at dose 0.6 mg/kg was 52 seconds, at 0.9 mg/kg was 44 seconds and at 1.2 mg/kg was 36 seconds).^23^,^33^,^45^–^52^

**Case 2: Septic shock**

You are caring for a 6-month-old unimmunized patient who presents with altered mental status and fever. Her initial vital signs are: HR 170 bpm, RR 52, BP 78/40, SpO2 92% on room air, with delayed capillary refill of 5 seconds. You place her on a NRB and give one push-pull bolus while ordering antibiotics. This improves her work of breathing and oxygenation, but her mental status and perfusion continue to worsen, and she develops worsening tachycardia, tachypnea, and her repeat blood pressure is 72/34. Your labs show a normal glucose, a procalcitonin of 20 ng/mL and a pH of 7.2. She is now responsive to painful stimuli only. You decide to intubate.

What are you concerned will happen when you intubate this patient**?**

How can you optimize this patient prior to intubation**?**

**Learning Points:**

- Hypotensive patients are at high risk for developing profound shock during RSI and are at particularly high risk for peri-intubation cardiac arrest. They may have both distributive shock as well as cardiac dysfunction in the setting of sepsis. 51,54
- Resuscitate before you intubate: 45–48,50, 54
  ○ In this patient, start a vasopressor before intubation (Epinephrine – can start at 0.05 or 0.1 mg/kg/hr).
  ○ Can give a second and third IVF push-pull bolus as well.
  ○ If in compensated shock, give push-pull boluses prior to intubation, and strongly consider starting a vasopressor before intubation.
  ○ Optimize preoxygenation (minimum of NRB+NC for apneic, ideally HFNC for preoxygenation and apneic oxygenation). These patients have very high oxygen consumption and low reserve and are at risk for rapid desaturation during intubation.
- All induction agents have the potential to cause hypotension. 50
  ○ Even ketamine**!** Ketamine increases sympathetic activity but does have a negative inotropic effect. Normally, the negative inotropic effect is outweighed by the catecholamine surge that increases heart rate and blood pressure, but in patients in shock, it is theorized that they may have already maximized their endogenous catecholamines, leading to predominance of the negative inotropic effect during RSI.
- Ketamine is still the most hemodynamically neutral drug we have for RSI and should be the induction drug of choice for pediatric shock. 50
  ○ It is reasonable to use a lower dose; can use 0.5–1 mg/kg (instead of the 2 mg/kg we use on the higher end of the spectrum). Some literature suggests even using < 0.5 mg/kg, but this is not standard practice. It is fine to still do the standard 1 mg/kg.
- Etomidate is contraindicated in pediatric sepsis (controversial). 46,48,50,51
- Fentanyl is reasonable, but not preferred over ketamine except in rare cases (infants < 3 months, congenital cardiac patients). 43,48,50
- Midazolam takes too long to take effect (~5 minutes) and is less hemodynamically favorable than ketamine. 48
- Due to poor perfusion, paralytics can take longer to take effect. To minimize this, use a dose at the higher end of the spectrum (1.5 mg/kg for rocuronium). Still reasonable to use 1.2mg/kg but would not go lower than that. 49
- If this patient presents to a large hospital with anesthesia/ENT backup, they may be a candidate for awake intubation in the OR if they are profoundly unstable despite resuscitation^42^,^45^
- Do not wait for these patients to be intubated in the PICU if you can appropriately and rapidly resuscitate them in the ED; they will only continue to decline. 41,45
- Anticipate the need to increase pressors post-intubation, if not already started. 51,54
- Two related points (if you have time): 45,51, 54
  ○ Hemorrhagic shock: Start with blood instead of fluid. If you have active bleeding, the patient**’**s shock is going to worsen until you get to the OR. You must intubate more rapidly than you do in medical patients. Get the blood in, consider starting an early vasopressor, and then intubate to get this patient to the OR. If you delay too long, they will only get worse.
  ○ New onset cardiogenic shock (eg, myocarditis, rapidly worsening congestive heart failure); if not forced to act, consider getting this patient to the cardiac ICU where they can be put onto extracorporeal membrane oxygenation (ECMO) +/− intubation.

**Case 3: Metabolic Acidosis**

You are caring for a 3-year-old patient who presents after an accidental metformin overdose. She is responsive to painful stimuli, with an initial HR of 145 bpm, SpO2 92%, RR 60, BP of 80/42, pH of 6.9, and lactate of 12 mmol/L. You are concerned that you will need to intubate her.

What are you concerned will happen if you intubate this patient**?**

How can you optimize this patient prior to intubation**?**

**Learning Points:**

- For patients in profound metabolic acidosis (pH < 7.1), respiratory compensation is the only thing keeping the patient**’**s pH from decreasing to levels incompatible with life. 45,46, 54
- These patients (eg, diabetic ketoacidosis [DKA], aspirin/metformin overdose, shock) may have extremely high minute ventilation that cannot be matched by the ventilator. Additionally, the apnea induced by RSI may be enough to cause rapid decompensation and cardiac arrest. 45,46, 54
- Additionally, metabolic acidosis causes vasodilation and cardiac dysfunction. 45,46,54
- If not forced to act, this patient could be a candidate for sedated (ketamine/dexmedetomidine) and topicalized/fiberoptic intubation in the OR with ENT/anesthesia. These patients may also be extracorporeal membrane oxygenation (ECMO) candidates. 45,46,50
- This patient will still need intubation for dialysis catheter placement and anticipated clinical course; however, some patients who are compensating appropriately for their metabolic acidosis can be managed medically (correcting DKA, etc.) if they are still protecting their airway despite their altered mental status. If the patient begins to also have respiratory failure or other event (seizure, etc.), you may be forced to intubate. 42,54
- If you must intubate this patient in the main ED or prior to transport (non-tertiary ED), do these things as rapidly as possible while preparing for intubation: 45,47,48,51–53
  ○ Fluid resuscitation – push-pull boluses.
  ○ Early vasopressors (in this patient, consider starting an epinephrine drip prior to intubation, have hung at bedside as a minimum).
  ○ Bicarbonate may not help these patients. While blood pH may briefly improve, it has not been shown to improve hemodynamics or change pH at the tissue level. Bicarbonate is metabolized carbon dioxide, which may worsen acidosis. However, some argue that at pH < 7.0, you are peri-arrest, so just give the bicarbonate.
  ○ Pre-oxygenation – HFNC+NRB, HFNC, or BiPAP (avoid if strong concern for emesis).
  ○ Most experienced intubator or intubator with very high chance of first pass success. Strongly consider anesthesia presence.
  ○ Use an RSI sedative that will preserve respiratory drive (ketamine).
  ○ Consider succinylcholine (if no contraindications) rather than rocuronium because this will restore spontaneous respiration faster.
  ○ Bag during RSI to prevent true apnea; allowing ANY true apnea time prior to laryngoscopy can crash this patient**’**s pH and lead to cardiac arrest.
  ○ Intubate as rapidly as possible.
- Post-intubation: 1,42,48
  ○ Minimize any apnea time while securing the tube.
  ○ You likely will not be able to match their pre-intubation minute ventilation, but remember to hyperventilate while manually bagging.
  ○ Check pre-intubation ETCO2 and try to match this post-intubation.
  ○ On the ventilator, will require a high respiratory rate (30–40+) with high tidal volumes (up to 8–10 cc/kg) to get as close to pre-intubation minute ventilation as possible without breath stacking. PICU can be an excellent resource for managing these incredibly sick patients.
  ○ Frequent blood gas testing and ventilator adjustment.
- For obtunded DKA patients with profound metabolic acidosis (your most likely category of severe metabolic acidosis patients): 41,45,52
  ○ As above, bicarbonate is not helpful, and additionally is contraindicated in DKA due to concern for cerebral edema.
  ○ Do your absolute best not to intubate them.
  ○ Focus all your efforts on reversing their DKA. If they are obtunded but still maintaining their respiratory compensation, focus on correcting their underlying physiology rather than the airway.

**Case 4: Delayed Sequence Intubation (DSI)**

You are caring for a 14-year-old patient with complex past medical history who presents with bilateral pneumonia after an aspiration event several days ago. HR 120 bpm, RR 30, BP 102/66, SpO2 70%. She is agitated. You attempt to place a non-rebreather on her face, but she rips it off. You are concerned you may need to intubate her.

What are you concerned will happen if you intubate this patient**?**

How can you optimize this patient prior to intubation**?**

**Learning Points:**

- This hypoxic patient is at high risk for profound desaturation (with subsequent potential cardiac arrest or hypoxic brain injury) during intubation without appropriate preoxygenation. 45–47
- Her agitation is preventing you from appropriate preoxygenation (as listed in scenario **#**1, NRB at flush rate + NC at 15 L/min or HFNC/BiPAP for at least 3 minutes in spontaneously breathing patients, or BVM with PEEP valve x 8 tidal volume breaths). This patient may benefit significantly from HFNC which can be used for both preoxygenation and apneic oxygenation. 45–47
- While immediately proceeding with RSI in this patient is dangerous, she is an excellent candidate for delayed sequence intubation (DSI). Instead of immediate RSI, first administer ketamine or dexmedetomidine at a sedation dose (administer as you do for sedation, not rapid push like for RSI). This will control the patient**’**s agitation and allow you to preoxygenate appropriately. Consider this procedural sedation where the procedure is preoxygenation. 45,50
- Ketamine and dexmedetomidine are ideal because they will preserve spontaneous breathing without the risks of etomidate (myoclonus/masseter spasm) or propofol (apnea, hypotension), not often used in pediatrics. 45,50
- After appropriate sedation and preoxygenation and patient positioning, you can administer your paralytic and proceed with intubation. 1,45,50
- There is a chance that after her agitation is controlled (ketamine, dexmedetomidine), and you place her on oxygen, she may not even need intubation. If the hypoxia was contributing significantly, and she settles out on HFNC, you could avoid intubation. 45,50
- For hypoxic smaller children, you may consider physical restraint for optimal preoxygenation. However, by using DSI and sedating them for preoxygenation, you reduce their oxygen consumption and improve their compliance with preoxygenation (better BiPAP seal, NRB not moving as patient flails their head around). 24,43,45,50
- What if this patient is obese**?** Review ramped positioning.^27^–^30^
  ○ Anatomic differences in the patient with BMI > 35:
    ▪ Increased airway resistance, elevated diaphragm, increased chest wall resistance, lower functional residual capacity (FRC), larger tongue, increased chance of pharyngeal wall collapse with lying flat and with RSI.
    ▪ Due to increased mass of the chest wall, as well as high prevalence of OSA, obese patients will have optimal oxygenation and ventilation in the upright or semi-upright position.
    ▪ May have worse glottic view, may benefit from a bougie or a D-blade.
  ○ Physiologic differences:
    ▪ Increased O2 consumption and CO2 production-> lower safe apnea time.
    ▪ RSI medication dosing in obese patients is very controversial, and there is almost no data in obese pediatric patients.
    ▪ If you dose opioids, benzodiazepines, or propofol based on TBW in patients with BMI >40, you can cause respiratory depression, apnea, and hypotension. If you dose your paralytic based on TBW, it will just last a lot longer. For your standard ketamine/rocuronium (dose based on IBW not TBW): for younger children, you can use the Broselow tape for a quick IBW. However, it should be noted that type of weight is still debated in the literature. For adolescents, unless extremely tall, you can just max out your doses at what they would be for a 70 kg patient.

**Case 5: Status asthmaticus**

You are caring for a 4-year-old patient who presents in status asthmaticus. Despite bronchodilators, steroids, ketamine, magnesium, and BiPAP, your patient is not improving. His respiratory rate is decreasing, and he has progressed to respiratory failure.

What are you concerned will happen if you intubate this patient**?**

How can you optimize this patient prior to intubation**?**

**Learning Points:**

- This patient is at risk for rapid hypoxic desaturation during intubation. Post-intubation, you are at risk for breath stacking and increased intrathoracic pressure causing decreased cardiac output and potential hemodynamic collapse. 45,53
- If a patient**’**s status asthmaticus is severe enough to intubate, you should give epinephrine prior to intubation: IM epinephrine + epinephrine drip. It is also reasonable to try terbutaline if readily available (unlikely) while also setting up for intubation. 45,50,53
- You are already preoxygenating with BiPAP + ketamine. Great**!** 47
- As above, make sure to have some form of apneic oxygenation for this patient and limit intubation duration. 47
- Consider succinylcholine as your paralytic; you can return them to spontaneous ventilation on the ventilator. 50
- Post-intubation: do not hyperventilate the patient. Significantly decrease respiratory rate when bagging and on the ventilator: half the normal respiratory rate with larger tidal volumes. 1
- Get them on the ventilator as soon as possible so you can see if they are breath stacking. 1
- Head of bed up to 30 degrees as soon as possible for better mechanics. 1
- On the ventilator: allow for permissive hypercapnia (pH down to 7.2) with vigilance to avoid breath stacking on the ventilator, and frequent blood gas analysis. Decrease your respiratory rate and have a prolonged inspiratory-to-expiratory (I:E) ratio. PICU can be an excellent resource here for managing the vent in these patients. 1,46
- Most pure respiratory acidosis patients are not a physiologically difficult intubation (for example, intubating an apneic post-ictal patient), because the ventilation is the problem, and you can start fixing it with PPV immediately and fix it more long-term with an ETT. In metabolic acidosis, ventilation is your compensation, not the problem, and you stop your compensation when you intubate. PPV will not help much if you are already at maximum minute ventilation compensating for a metabolic acidosis. Pure respiratory acidosis is also unlikely to lead to very profound acidosis (pH < 6.9). 45,46
- Pure respiratory acidosis patients are good candidates for DSI: ketamine/dexmedetomidine + BiPAP to start improving acidosis prior to intubation. Often, the patient even wakes up when their pH improves and you may be able to avoid intubation. 45,46

*Pediatric Difficulty Airway Day Clinical Pearls*

**Neonatal airway**

- Neonatal airways have many anatomic and physiologic differences compared to airways of older children or adults. 20–24
  ○ Bradycardia is primarily driven by hypoxia in this population
  ○ Neonates have different saturation goals based on minute and hour of life
  ○ Prior to considering intubation, corrective steps should be completed
- MR SOPA is an acronym that can be used for the ventilation corrective steps:^22^,^24^
  ○ Mask Readjustment
  ○ Reposition Airway
  ○ Suction mouth and nose
  ○ Open mouth
  ○ Positive pressure
  ○ Alternative airway
- LMAs can be used in infants 2 kg or greater (approximately corresponding to a 34-week infant). 22,24
- Intubation is completed without RSI medications.^22^,^25^
- Neonatal ETT size and blade size is based on gestational age and weight.^22^
  ○ <28 weeks gestation: 2.5 endotracheal tube (ETT) and 00 or 0 blade
  ○ 28–34 weeks gestation: 3.0 ETT and 0 blade
- Intubation attempts should be limited to 30–45 seconds in neonates due to low reserves.^22^,^25^
- Lip lift will help to increase space in the oral cavity.^22^,^24^

**Obese patient airway**

- Patients with BMI>35 have anatomic and physiologic differences and are likely to desaturate rapidly.^26^–^28^
- Optimal pre-oxygenation is in the upright or semi-upright position.^28^–^30^
- If possible, consider pre-oxygenation with high flow nasal cannula (HFNC) or BiPAP.^29^,^30^
- Optimal head positioning is in ear to sternal notch position.^28^–^30^
- RSI dosing is controversial, but consider using IBW for initial medication dosing.^26^–^28^
- Consider use of a hyperregulated blade for improved glottic views.^27^,^28^
- Tidal volume should be based on IBW not TBW.^26^–^28^

**Inhalational injuries**

- Inhalational injuries are often from exposure to thermal injury. Patients who are trapped in enclosed spaces (eg, house fire requiring extrication) are at higher risk. 33–35
- Upper airway injury signs: discoloration, soot in airway, stridor, hoarse voice. 33–35
- Lower airway injury signs: coughing, wheezing, or dyspnea. 33–35
- Progressive airway injury occurs over 24–48 hours. 33–35
- Early intubation for patients at high risk for progressive injury is the mainstay treatment for inhalational injuries. 33–35
- In the stable patient, can consider bedside scope to evaluate extent of inhalational injury prior to determining airway management strategy. 33–35
- Consider racemic epinephrine and/or albuterol while preparing for intubation. 33–35
- Consider pre-oxygenation with HFNC if possible. 33–35
- Presume carbon monoxide poisoning until excluded and treat with 100% oxygen. 33–35
- When considering ETT size, it is good practice to have ETT of estimated size and up to 2 sizes smaller in the case of extensive airway edema. 33–35
- Consider calling the difficult airway team if available. 33–35
- Back-up airway will be supraglottic airway, but be prepared for a surgical airway.^33^–^35^

**Contaminated airway**

- Airways can be contaminated by various substances such as emesis, pulmonary edema, secretions, or blood, which can make visualization difficult.^23^,^33^,^37^
- Suctioning optimization will help with intubation success.^39^
  ○ Double suction
  ○ Large bore suction
  ○ Using ETT as suction
  ○ Suction assisted laryngoscopy and airway decontamination (SALAD) technique
- Consider airway adjuncts, such as use of a bougie, to optimize success.^23^,^33^,^37^,^38^
- Supraglottic airway or bag-mask ventilation may not be successful if significant contamination.^23^,^33^,^37^,^38^

**Airway management in the physiologically difficult patient**

- Three main buckets for the physiologically difficult airway: shock, hypoxia, and metabolic acidosis. There can be significant overlap.^45^
- Mnemonic = HOP Killers (Hypotension, Oxygenation, pH) 54
- Predictors of peri-arrest intubation in pediatric intubation: hypotension, myocardial dysfunction, persistent hypoxemia, severe metabolic acidosis (pH < 7.1), post-return of spontaneous circulation (ROSC), and status asthmaticus. 45, 51
- Most pure respiratory acidosis patients are not a physiologically difficult intubation (for example, intubating an apneic post-ictal patient), because the ventilation is the problem, and you can start fixing it with PPV immediately and fix it more long-term with an ETT. In metabolic acidosis, ventilation is your compensation, not the problem, and you stop your compensation when you intubate. PPV will not help much if you are already at maximum minute ventilation compensating for a metabolic acidosis. Pure respiratory acidosis is also unlikely to lead to very profound acidosis (pH < 6.9). 45,47
- Resuscitate before you intubate. You are rarely forced to act immediately. 45,47, 51
- If this patient comes to a community ED, consider calling transport as soon as possible to get to tertiary care center. For these stations, there is generally no one right answer. But there is a wrong answer, which is RSI before resuscitation/optimization. 42
- If a patient’s anticipated course is deterioration, often the sooner you can intubate after appropriate resuscitation, the better. 42

**Pediatric airway adjuncts**

- Failed airway: Failure to maintain oxygenation or ≥ 3 attempts by an experienced operator. 33,41,42
- Adjunct options: oropharyngeal airway, supraglottic airway, bougie, hyperangulated blade^23^,^33^,^41^,^43^
- The Storz hyperangulated pediatric blade is typically too large for neonatal/infant airways^1^,^21^,^42^,^44^–^48^
- 4.0 ETT is the smallest that can fit a pediatric bougie^40^

## SMALL GROUPS LEARNING MATERIALS

### Appendix B Summary of List of Equipment Needed for All Scenarios

**Table:**

**Setting:** ☒ Clinic □ Inpatient □ Critical Care ☒ Emergency Department □ OR □ PACU □ OB/GYN □ Behavioral Health □ Task training □ Home environment □ Virtual/Augmented reality □ Outreach: □ OTHER:	**Manikin/Task Trainer:** ☒ Gaumard Premie □ Gaumard Super Tory □ Gaumard Newborn □ Gaumard 1 year-old □ Gaumard 5 year-old □ Gaumard Advanced 5 year-old □ Gaumard Victoria □ Laerdal SimBaby □ Laerdal SimMan 3G ☒ Laerdal SimMan Essentials □ Low fidelity 5 year-old □ Trach Rolling Refresher Cart □ Trach Rolling Refresher Cart □ CPR Rolling Refresher Cart □ ASL 5000 □ Trauma Child □ Vascular Access Child □ Lumbar Puncture Baby (Qty: ) □ Intubation head (Qty: I, **P**, A ) ☒ OTHER: Burn manikin, emesis manikin	**Equipment Attached to Manikin** (specify location if applicable)**:** □ IV fluids: □ IV meds: ☒ Oxygen: Via Rac epi nebulizer, NRB, NC ☒ PIV(s) □ EKG leads ☒ Pulse-ox ☒ Monitor □ Arterial line □ Central line ☒ ETT (size: , marking: ): cuffed 2.0, 2.5, 3.0, 7.0, 7.5, 8.0; uncuffed 3.5 uncuffed; ETT stylet □ Ventilator: □ Trach (size: , type: ) □ Trach bag □ NG/OG □ Training defib pads (□ Zoll Defib snaps) □ C-collar: ☒ Moulage (specific details and recipe if applicable). ☒ OTHER: Bandaid, pillow padding to simulate an obese body habitus, moulage, fake emesis

**Table:**

**Equipment Available in Room:** ☒ Crash cart ☒ Zoll ☒ Training defib pads (□ Zoll Defib snaps) ☒ BVM (size: ☒ neo ☒ ped, ☒ adult) ☒ Monitor ☒ IV start kit and tubing (Qty: 4 ) □ IV pump (□ infusion (#),□ syringe (#), □ brain (#)) □ Push-pull supplies: ☒ IO access supplies: □ Rapid infuser □ Epi Auto-injector (□ junior (#, type) □ adult (#, type)) ☒ Intubation supplies: Miller 00, 0, 1, 2; Mac 2, 3, 4; ETT – cuffed 2.0, 2.5, 3.0, 3.5, 4.0, 4.5, 7.0, 7.5, 8.0; uncuffed 3.5 uncuffed; ETT stylet ☒ C-MAC: Miller 0, 1, 2; Mac 0, 2, 3, 4; adult D-blade ☒ Oxygen device(s): nasal cannula, NeoPuff, HFNC, NRB, NC □ Aerosol device: ☒ Suction device(s): flexible catheter, Yankauer (x4) ☒ NP/OP/LMAs: neonatal; LMA 2, 2.5, 4; adult NP/OP □ Ventilator: □ Chest tube kit: ______ □ Central line kit: □ Cricothyrotomy kit: □ Surgical equipment: ☒ Stethoscope □ Task trainer: □ C-collar □ Vocera ☒ Emergency med sheet: 1 kg , 15 kg, 55 kg ☒ OTHER: Blankets for ramping patient, adult bougie	**Medications and fluids:** ☒ IV fluids: NS/LR ☒ IV meds: epinephrine, RSI (Ketamine, Rocuronium), albuterol □ Oral meds: □ IM / SC meds: ☒ Inhalation meds: Racemic epinephrine, albuterol **Diagnostics Available:** □ Labs: ☒ Imaging: CXR (shows signs of RDS), CXR (shows PNA, ETT in appropriate position) □ 12 lead EKG □ OTHER: **Room Set-up Comments:** **Additional Information:**

### Appendix C Pre-Survey

1. Please restate your unique identifier that combines the following in order: ________________________
  First two letters of your mother's first name Date of birth (February 8th = “08”, not “8”) First two letters of the town you were born in
  For example, “Patricia” “13th” “Minneapolis” -> “PA13MI”
2. What is your current year?
  **Table:**
  PGY1	PGY2	PGY3	Fellow	Attending
3. If you are a first-year fellow, have you completed your anesthesia rotation? Yes No

Please rate your current ability to perform each of the following tasks:

**Table:**

	Could not perform	May be able to perform with assistance	May be able to perform independently	Easily able to perform independently
4. Direct laryngoscopy with intubation				
5. Video laryngoscopy with intubation				
6. LMA Placement				
7. Bougie-assisted intubation				
8. Video laryngoscopy with hyperangulated laryngoscope blade with intubation				
9. Explain the options and techniques for managing the anatomically difficult airway				
10. Explain the options and techniques for managing the physiologically difficult airway				
11. Describe management of the failed airway				

12. What Cormack-Lehane view is this?
  - 1
  - 2a
  - 2b
  - 3
  - 4
13. What Cormack-Lehane view is this?
  - 1
  - 2a
  - 2b
  - 3
  - 4
14. What is the lower weight limit for a size 1 LMA?
  - 1 kg
  - 2 kg
  - 3 kg
  - 4 kg
15. What is the smallest size ETT you can fit over a pediatric size bougie?
  - 3.5
  - 4.0
  - 4.5
  - 5.0
  - 6.0
16. What are indications for use of the pediatric D-blade? (circle all that apply)
  - Contaminated airway
  - Tongue edema
  - Hypoxia
  - Limited mouth opening
  - Neck immobilization
17. Which laryngoscope blade is appropriate for intubating a 30-week estimated gestational age newborn? (circle all that apply)
  - Miller 00
  - Miller 0
  - Miller 1
  - Mac 0
  - Mac 1
18. What is an appropriate sedative medication for RSI in a newborn? (circle all that apply)
  - Atropine
  - Etomidate
  - Fentanyl
  - Ketamine
  - Midazolam
19. You are intubating an 8-month-old infant with bronchiolitis. On your first 45 second attempt at intubation, you see a Cormack-Lehane Grade 3 view. Your RT administers BVM ventilations before your next attempt, but after 30 seconds, the patient’s SpO2 remains at 75%, and you see minimal chest rise with breaths. What is the next best step?
  - Continue bagging the patient
  - Perform an immediate second intubation attempt with the most experienced intubator
  - Insert an LMA and continue ventilation prior to a second intubation attempt
  - Perform a needle cricothyroidotomy
20. An unimmunized 3-year-old presents with respiratory distress, stridor, and drooling. He is hypoxic on arrival, and rapidly becomes unresponsive and apneic but has a faint pulse. You see no chest rise with a BVM. You attempt to intubate, but are unsuccessful due to severe edema of the epiglottis. What is the next best step?
  - Continue bagging the patient using a BVM
  - Perform an immediate second intubation attempt with the most experienced intubator
  - Insert an LMA and continue ventilation prior to a second intubation attempt
  - Perform a needle cricothyroidotomy
21. What is the SALAD technique?
  - An approach to managing intubation for the patient with a contaminated airway
  - An approach to managing intubation for the patient with hypotension
  - An approach to managing intubation for the post-ROSC patient
  - An approach to managing intubation for the patient with severe metabolic acidosis
22. What modifications can you perform when intubating an obese patient? (circle all that apply)
  - Preoxygenation with HFNC or BiPAP
  - Positioning the patient in a ramped position
  - Dosing ketamine based on total body weight
  - Starting vasopressors prior to intubation
  - Use of apneic oxygenation with nasal cannula or HFNC
23. What modifications can you perform when intubating patients with inhalational injuries? (circle all that apply)
  - Administer albuterol
  - Administer racemic epinephrine
  - Prepare ETT up to 2 sizes smaller than appropriate for weight
  - Call anesthesia/ENT to discuss fiberoptic intubation in the OR
24. Which patient(s) are physiologically difficult intubations? (circle all that apply)
  - Metabolic acidosis
  - Hypotension
  - Contaminated airway
  - Persistent hypoxia
  - Respiratory acidosis
  - Post-ROSC
25. A 12-year-old patient presents in DKA and septic shock due to pneumonia. Her pH is 6.95. She has received 1L IV fluids, and has been started on insulin and broad-spectrum antibiotics. While in your ED, she becomes progressively obtunded, and has worsening respiratory effort. The decision is made to intubate. Her heart rate is 120 and her blood pressure is 82/34. What steps can you take prior to intubation? (circle all that apply)
  - Consider preoxygenation with HFNC or BiPAP
  - Initiate vasopressors
  - Administer another 1L fluid bolus
  - Administer sodium bicarbonate
26. Despite your efforts, the patient’s repeat blood pressure prior to intubation is 78/34. What sedative medication and dose is appropriate for RSI (circle all that apply)
  - Ketamine 1 mg/kg
  - Ketamine 0.5 mg/kg
  - Etomidate 0.3 mg/kg
  - Propofol 2 mg/kg
  - Fentanyl 3 mcg/kg
27. Which of these situation(s) is a failed airway? (circle all that apply)
  - Failure of the first intubation attempt
  - Failure of three intubation attempts
  - Failure of three intubation attempts by an experienced operator
  - Failure of the first intubation attempt, with subsequent failure to maintain SpO2 ≥ 90%

### Appendix D Post-Survey

1. Please restate your unique identifier that combines the following in order: ________________________
  First two letters of your mother's first name Date of birth (February 8th = “08”, not “8”) First two letters of the town you were born in
  For example, “Patricia” “13th” “Minneapolis” -> “PA13MI”

Please rate your current ability to perform each of the following tasks:

**Table:**

	Could not perform	May be able to perform with assistance	May be able to perform independently	Easily able to perform independently
2. Direct laryngoscopy with intubation				
3. Video laryngoscopy with intubation				
4. LMA Placement				
5. Bougie-assisted intubation				
6. Video laryngoscopy with hyperangulated laryngoscope blade with intubation				
7. Explain the options and techniques for managing the anatomically difficult airway				
8. Explain the options and techniques for managing the physiologically difficult airway				
9. Describe management of the failed airway				

10. What Cormack-Lehane view is this?
  - 1
  - 2a
  - 2b
  - 3
  - 4
11. What Cormack-Lehane view is this?
  - 1
  - 2a
  - 2b
  - 3
  - 4
12. What is the lower weight limit for a size 1 LMA?
  - 1 kg
  - 2 kg
  - 3 kg
  - 4 kg
13. What is the smallest size ETT you can fit over a pediatric size bougie?
  - 3.5
  - 4.0
  - 4.5
  - 5.0
  - 6.0
14. What are indications for use of the pediatric D-blade? (circle all that apply)
  - Contaminated airway
  - Tongue edema
  - Hypoxia
  - Limited mouth opening
  - Neck immobilization
15. Which laryngoscope blade is appropriate for intubating a 30-week estimated gestational age newborn? (circle all that apply)
  - Miller 00
  - Miller 0
  - Miller 1
  - Mac 0
  - Mac 1
16. What is an appropriate sedative medication for RSI in a newborn? (circle all that apply)
  - Atropine
  - Etomidate
  - Fentanyl
  - Ketamine
  - Midazolam
17. You are intubating an 8-month-old infant with bronchiolitis. On your first 45 second attempt at intubation, you see a Cormack-Lehane Grade 3 view. Your RT administers BVM ventilations before your next attempt, but after 30 seconds, the patient’s SpO2 remains at 75%, and you see minimal chest rise with breaths. What is the next best step?
  - Continue bagging the patient
  - Perform an immediate second intubation attempt with the most experienced intubator
  - Insert an LMA and continue ventilation prior to a second intubation attempt
  - Perform a needle cricothyroidotomy
18. An unimmunized 3-year-old presents with respiratory distress, stridor, and drooling. He is hypoxic on arrival, and rapidly becomes unresponsive and apneic but has a faint pulse. You see no chest rise with a BVM. You attempt to intubate, but are unsuccessful due to severe edema of the epiglottis. What is the next best step?
  - Continue bagging the patient using a BVM
  - Perform an immediate second intubation attempt with the most experienced intubator
  - Insert an LMA and continue ventilation prior to a second intubation attempt
  - Perform a needle cricothyroidotomy
19. What is the SALAD technique?
  - An approach to managing intubation for the patient with a contaminated airway
  - An approach to managing intubation for the patient with hypotension
  - An approach to managing intubation for the post-ROSC patient
  - An approach to managing intubation for the patient with severe metabolic acidosis
20. What modifications can you perform when intubating an obese patient? (circle all that apply)
  - Preoxygenation with HFNC or BiPAP
  - Positioning the patient in a ramped position
  - Dosing ketamine based on total body weight
  - Starting vasopressors prior to intubation
  - Use of apneic oxygenation with nasal cannula or HFNC
21. What modifications can you perform when intubating patients with inhalational injuries? (circle all that apply)
  - Administer albuterol
  - Administer racemic epinephrine
  - Prepare ETT up to 2 sizes smaller than appropriate for weight
  - Call anesthesia/ENT to discuss fiberoptic intubation in the OR
22. Which patient(s) are physiologically difficult intubations? (circle all that apply)
  - Metabolic acidosis
  - Hypotension
  - Contaminated airway
  - Persistent hypoxia
  - Respiratory acidosis
  - Post-ROSC
23. A 12-year-old patient presents in DKA and septic shock due to pneumonia. Her pH is 6.95. She has received 1L IV fluids, and has been started on insulin and broad-spectrum antibiotics. While in your ED, she becomes progressively obtunded, and has worsening respiratory effort. The decision is made to intubate. Her heart rate is 120 and her blood pressure is 82/34. What steps can you take prior to intubation? (circle all that apply)
  - Consider preoxygenation with HFNC or BiPAP
  - Initiate vasopressors
  - Administer another 1L fluid bolus
  - Administer sodium bicarbonate
24. Despite your efforts, the patient’s repeat blood pressure prior to intubation is 78/34. What sedative medication and dose is appropriate for RSI (circle all that apply)
  - Ketamine 1 mg/kg
  - Ketamine 0.5 mg/kg
  - Etomidate 0.3 mg/kg
  - Propofol 2 mg/kg
  - Fentanyl 3 mcg/kg
25. Which of these situation(s) is a failed airway? (circle all that apply)
  - Failure of the first intubation attempt
  - Failure of three intubation attempts
  - Failure of three intubation attempts by an experienced operator
  - Failure of the first intubation attempt, with subsequent failure to maintain SpO2 ≥ 90%
  **Table:**
  	Strongly Disagree	Disagree	Neutral	Agree	Strongly Agree
  26. The premature neonate station added to my pediatric airway knowledge and skills					
  27. The failed airway station added to my pediatric airway knowledge and skills					
  28. The contaminated airway station added to my pediatric airway knowledge and skills					
  29. The burn airway station added to my pediatric airway knowledge and skills					
  30. The physiologically difficult airway station added to my pediatric airway knowledge and skills					
  31. The obese patient station added to my pediatric airway knowledge and skills					
  32. The PEM difficult airway course added to my pediatric airway knowledge and skills					
33. List one “take away” that you will apply when taking care of patients in the future.
34. List one airway management topic you still feel uncomfortable.

### Appendix E Answer Key

1. What Cormack-Lehane view is this?
  - 1
  - 2a
  - 2b
  - 3
  - 4
2. What Cormack-Lehane view is this?
  - 1
  - 2a
  - 2b
  - 3
  - 4
3. What is the lower weight limit for a size 1 LMA?
  - 1 kg
  - 2 kg
  - 3 kg
  - 4 kg
4. What is the smallest size ETT you can fit over a pediatric size bougie?
  - 3.5
  - 4.0
  - 4.5
  - 5.0
  - 6.0
5. What are indications for use of the pediatric D-blade? (circle all that apply)
  - Contaminated airway
  - Tongue edema
  - Hypoxia
  - Limited mouth opening
  - Neck immobilization
6. Which laryngoscope blade is appropriate for intubating a 30-week estimated gestational age newborn? (circle all that apply)
  - Miller 00
  - Miller 0
  - Miller 1
  - Mac 0
  - Mac 1
7. What is an appropriate sedative medication for RSI in a newborn? (circle all that apply)
  - Atropine
  - Etomidate
  - Fentanyl
  - Ketamine
  - Midazolam
8. You are intubating an 8-month-old infant with bronchiolitis. On your first 45 second attempt at intubation, you see a Cormack-Lehane Grade 3 view. Your RT administers BVM ventilations before your next attempt, but after 30 seconds, the patient’s SpO2 remains at 75%, and you see minimal chest rise with breaths. What is the next best step?
  - Continue bagging the patient
  - Perform an immediate second intubation attempt with the most experienced intubator
  - Insert an LMA and continue ventilation prior to a second intubation attempt
  - Perform a needle cricothyroidotomy
9. An unimmunized 3-year-old presents with respiratory distress, stridor, and drooling. He is hypoxic on arrival, and rapidly becomes unresponsive and apneic but has a faint pulse. You see no chest rise with a BVM. You attempt to intubate, but are unsuccessful due to severe edema of the epiglottis. What is the next best step?
  - Continue bagging the patient using a BVM
  - Perform an immediate second intubation attempt with the most experienced intubator
  - Insert an LMA and continue ventilation prior to a second intubation attempt
  - Perform a needle cricothyroidotomy
10. What is the SALAD technique?
  - An approach to managing intubation for the patient with a contaminated airway
  - An approach to managing intubation for the patient with hypotension
  - An approach to managing intubation for the post-ROSC patient
  - An approach to managing intubation for the patient with severe metabolic acidosis
11. What modifications can you perform when intubating an obese patient? (circle all that apply)
  - Preoxygenation with HFNC or BiPAP
  - Positioning the patient in a ramped position
  - Dosing ketamine based on total body weight
  - Starting vasopressors prior to intubation
  - Use of apneic oxygenation with nasal cannula or HFNC
12. What modifications can you perform when intubating patients with inhalational injuries? (circle all that apply)
  - Administer albuterol
  - Administer racemic epinephrine
  - Prepare ETT up to 2 sizes smaller than appropriate for weight
  - Call anesthesia/ENT to discuss fiberoptic intubation in the OR
13. Which patient(s) are physiologically difficult intubations? (circle all that apply)
  - Metabolic acidosis
  - Hypotension
  - Contaminated airway
  - Persistent hypoxia
  - Respiratory acidosis
  - Post-ROSC
14. A 12-year-old patient presents in DKA and septic shock due to pneumonia. Her pH is 6.95. She has received 1L IV fluids, and has been started on insulin and broad spectrum antibiotics. While in your ED, she becomes progressively obtunded, and has worsening respiratory effort. The decision is made to intubate. Her heart rate is 120 and her blood pressure is 82/34. What steps can you take prior to intubation? (circle all that apply)
  - Consider preoxygenation with HFNC or BiPAP
  - Initiate vasopressors
  - Administer another 1L fluid bolus
  - Administer sodium bicarbonate
15. Despite your efforts, the patient’s repeat blood pressure prior to intubation is 78/34. What sedative medication and dose is appropriate for RSI (circle all that apply)
  - Ketamine 1 mg/kg
  - Ketamine 0.5 mg/kg
  - Etomidate 0.3 mg/kg
  - Propofol 2 mg/kg
  - Fentanyl 3 mcg/kg
16. Which of these situation(s) is a failed airway? (circle all that apply)
  - Failure of the first intubation attempt
  - Failure of three intubation attempts
  - Failure of three intubation attempts by an experienced operator
  - Failure of the first intubation attempt, with subsequent failure to maintain SpO2 ≥ 90%
